# Supplementary material for: Health equity and wellbeing among older people’s caregivers in New Zealand during COVID-19: Protocol for a qualitative study
Source: PLoS One. 2022 Jul 15;17(7):e0271114. doi: 10.1371/journal.pone.0271114 (PMC9286244; doi:10.1371/journal.pone.0271114)
Supplement: S1 Appendix — (PDF) [file pone.0271114.s001.pdf]

School of Nursing
Faculty of Medical and Health Sciences
Building 505, 85 Park Road, Grafton
Tel: +64 9 373 7599 ext. 83990

Health Equity & Wellbeing among older people's caregivers during COVID 19 restrictions
Demographic Questions: Caregivers

Demographic Questions: Caregivers Study ID # : _________ multiple caregivers #:______

1. How old are you? (in years) _____________

2. To which gender identity do you most identify? ☐ Male ☐ Female ☐ Gender diverse

3. What is your current marital status? ☐ Married ☐ De Facto ☐ Divorced ☐ Separated ☐ Widowed ☐ Never married

4. To which ethnicity do you identify? Please tick all that apply, and if possible, circle one that you most identify with
   ☐ Māori ☐ NZ European ☐ Samoan ☐ Cook Island Māori
   ☐ Tongan ☐ Niuean ☐ Fijian ☐ Chinese
   ☐ Indian ☐ Other ________

5. What is your first or home language? _____________

6. What is your **highest** level of schooling you achieved? (tick only one)
   ☐ Primary (or less) ☐ High School (not completed) ☐ High School (completed) ☐ Trade
   ☐ University or Polytec undergraduate ☐ University postgraduate
