# Supplementary material for: Health equity and wellbeing among older people’s caregivers in New Zealand during COVID-19: Protocol for a qualitative study
Source: PLoS One. 2022 Jul 15;17(7):e0271114. doi: 10.1371/journal.pone.0271114 (PMC9286244; doi:10.1371/journal.pone.0271114)
Supplement: S2 Appendix — (PDF) [file pone.0271114.s002.pdf]

Health Equity & Wellbeing among older people's caregivers during COVID 19 restrictions
Semi-structured Interview Guide for Individual Caregivers

PREAMBLE: **We are interested in the types of support and care that you provided to an older person at the different alert levels during the COVID19 pandemic and whether this was same or different before the pandemic. We would like to discuss your experiences of caregiving and the resources that you drew on to support your role. We would also like to talk about how you managed any other roles that you had (other than caregiver). I'll refer to alert levels 1-4 as 'COVID19 restrictions'. Sometimes I will ask about particular alert levels.**
[SHOW ALERT LEVEL CARD, AND ESTABLISH THAT THE PARTICIPANT UNDERSTANDS Level 4, lockdown; Level 3, restrict; Level 2, reduce; Level 1, prepare]

You are taking part in this study because you said that you provided care or support to an older person during COVID19 restrictions. During levels 3 and 4 the Government referred to "bubbles". At Level 4 lockdown these were made up of the people you were living with. For most, people this was just the people in the household, but for some people, a bubble covered more than one household, for example, when someone was providing care to someone in a different household. At Alert Level 3 people were asked to remain in their household bubble whenever they were not at work or school, but the bubble could be expanded to connect with close family and whānau, bring in caregivers, or support isolated people.

*PURPOSE: The **individual** area of enquiry is structured to establish changes in caregiving during COVID-19 restrictions and positive and negative outcomes on personal resources/wellbeing. In addition to balancing work and care to an older person, caregivers may have faced additional child-care pressures exacerbated by school and nursery closures. Employers or business owners may have encountered additional stress associated with economic uncertainty.*	
**Question**	**Prompts**
Please tell me about the person or people that you provided care to during COVID19 restrictions? **NB: If caring for more than one person please ask participants to select/focus on one (or one group e.g., if it's two parents in a household) for remainder of questions.**	Age, relationship, and why they needed care/support, was care needed because the person had COVID19 (and if so was the caregiver tested).
Please tell me whether you provided support/care before COVID and whether it changed during COVID19 restrictions; or whether you started providing support/care during this period?	Changes in amount (hours per week or day) OR types of emotional/functional support changed OR living arrangements changed and why.
SHOW BUBBLE PICTURE CARDS: Please describe who was in your bubble at Level 4 and at Level 3?	Age and relationship of people in bubble at each level (did this always include care recipient?), number of households, proximity of other households (if any).
Please describe how the decisions about "who is in our bubble?" were made?	Were decisions easy to make, or tricky.
Please describe your typical day as a caregiver during COVID19 restrictions?	Routines, self-care, exercise, leisure, cooking, sleep.
How did you feel about caregiving during COVID19 restrictions make you feel?	Positive and negative aspects, health, emotional wellbeing
Did caregiving during COVID19 restrictions result in any additional out-of-pocket expenditure for you (compared to before this period), and if so, could you tell me what this was for? **NB we are not asking about $ amount**	Services, goods (e.g. bedding, mobility aids and incontinence products), home adaptations to improve accessibility, increased heating, laundry, transportation.
ASK PARTICIPANTS WHO EXPERIENCED ADDITIONAL EXPENDITURE ONLY:<br>How , if at all, did this increased out-of-pocket expenditure impact on you?	Financially, mental health
ASK ALL: Please describe any other roles and responsibilities that you had during lockdown, and how you managed these in relation to caregiving.	Relationships roles (e.g. spouse, mother, grandmother); employment; community or voluntary roles.
ASK PARTICIPANTS THAT WERE IN PAID EMPLOYMENT ONLY. SHOW ESSENTIAL WORKER PICTURE CARD: Please tell me more about your experience as an employee and caregiver, and whether this changed during COVID19 restrictions?	Type of work and hours worked; essential worker strategies for protecting the care-recipient from COVID19; flexible working at home; loss of job or primary source of income and any additional challenges this created

**<u>PREAMBLE:</u> Now I'd like to talk about your relationships with whānau, family, friends and neighbours (not paid/formal carers) and whether COVID19 restrictions impacted on your relationships, and influenced caregiving in any way.**	
*<u>PURPOSE</u>: This section will explore **family, social networks, interpersonal relationship** in relation to caregiving. Whereas large, integrated networks are more easily "activated" to protect individuals in the face of crisis, disjointed or attenuated networks may not have been so responsive. Family and caregiving networks may have been hampered by social distancing. The caregiver-care recipient relationship may have been strained due to increases in caregiving tasks, hyper-vigilance, and less respite.*	
**Question**	**Prompts**
Please tell me about your personal relationship with [care recipient] during COVID19 restrictions, for example, was it the same as before restrictions or did it change?	Changes across alert levels, the quality of relationship
Please tell me whether any whānau, family, friends and neighbours helped you to provide support/care during the COVID19 restrictions?	For each: who (relationship to caregiver/care-recipient), care/support provided
ASK PARTICIPANTS THAT IDENTIFIED OTHER CAREGIVERS :<br>Please tell me a little bit more about whether [each person] provided support/care before COVID19 restrictions and whether it changed during restrictions; or whether they started providing support/care during this period.	Changes in amount (hours per week or day) OR types of emotional/functional support changed OR living arrangements changed and why. AND challenges/barriers to coordinating caregiving activities.
ASK PARTICIPANTS THAT IDENTIFIED OTHER CAREGIVERS :<br>How did you co-ordinate activities between you?	
ASK PARTICIPANTS THAT IDENTIFIED NEW CAREGIVERS DURING COVID19 RESTRICTIONS<br>Please describe whether the new caregivers continued to help beyond levels 3 and 4 and what you feel about this?	Feeling more or less connected / supported.
ASK ALL<br>Please tell me whether any whānau, family, friends and neighbours supported you (rather than the care recipient) during COVID19 restrictions?	For each: who (relationship to caregiver), care/support provided
Please tell me about the quality of your other personal relationships during COVID19 restrictions, for example, were they the same as before lockdown or did they change?	Why; how did it make the participant feel?

**<u>PREAMBLE</u>: Next, I would like to discuss any contact that you had with organisations that provided you (or the care recipient) with care or support during COVID19 restrictions, for example, GP, district nurses, local health or social care providers, iwi, charitable or faith-based organisations**	
*<u>PURPOSE:</u> The **organisational** area of enquiry will establish whether clinical home care service guidelines need updating in light of experiences during the pandemic. For example, how did strategies developed for formal home care providers during COVID-19 to protect staff and organisational integrity, alongside issues concerning the availability of resources (e.g. PPE, respite, day care), impact on caregivers? Did the application of DHB's telemedicine and telehealth support or hinder caregivers?*	
**Question**	**Prompts**
Please tell me which, if any, formal service providers, organisations, groups, marae, churches or schools supported you or provided care to [care recipient(s)] during the COVID19 restrictions? Who were they and how did they help?	Identify each provider (and type of organisation) and the care/support provided, material resources (e.g., food, supplies), COVID19 testing, cleaning, home modification, equipment installation. In-person visits, digital connection (e.g. telemedicine, telehealth) or telephone.
ASK IF FORMAL SERVICES USED, FOR **EACH** ORGANISATION:<br>Please tell me a little bit more about whether [each organisation] provided support/care before COVID19 restrictions and whether it changed during restrictions; or whether they started providing support during this period?	Changes in amount (hours per week or day) OR types of care support changed and why.
ASK IF NEW FORMAL SERVICES/SUPPORT PROVIDED:<br>Please describe whether the new services or support continued beyond levels 3 and 4 and what you feel about this?	Feeling more or less connected / supported.
ASK IF FORMAL SERVICES USED, FOR **EACH** ORGANISATION:<br>Please tell me about your experiences of the support or services that you received and the relationships you had with the staff or people providing help.	Changes in relationships e.g. managing PPE issues while protecting care-recipient from COVID19 with a trusted/long-term home care worker, issues for ongoing relationships. Turnover of staff, negotiating new relationships with new workers. Satisfaction with telehealth/telemedicine.
ASK IF FORMAL SERVICES USED:<br>How were these services coordinated?	Key worker, caregiver, health navigator, easy/difficult
Did you have to solve any problems associated with managing health, care or other practical things associated with caregiving (such as shopping) during COVDI19 restrictions? If so, please describe in as much detail as possible, how you found the information you needed	What did they do/who did they ask, online/telephone/in person. Culturally or linguistically safe, appropriate and accessible. Generic resources suitable for people living with dementia. Level of confidence in the information provided.

Please describe any challenges associated with getting or receiving help from organisations during the COVID19 restrictions?	Difficulties with navigating health and social care systems, communication, co-ordination or delivery (e.g. no PPE), concerns about letting care workers into home including increased stress, digital access, appointments with GPs and specialists, flexibility , additional roles taking up slack where there were gaps in support / services.
Please describe anything you think should have been provided during COVID19 restrictions, that wasn't?	GP practice, pharmacy, normally scheduled services, respite, relevance or services, socially, culturally or linguistically appropriate and accessible
Please describe any new ways in which organisations provided information or support that worked really well for you?	Innovations for routine use beyond restrictions

**PREAMBLE : Now I want to ask you about the community and neighbourhood you lived in during COVID19 restrictions – the local amenities that were available to you, what sort of vibe it had and the interactions you had within it.**	
PURPOSE: *The **community/ neighbourhood** area of enquiry will identify inequities in access to services for caregivers in disadvantaged communities and for those living at a distance from 'resource centres'. We also need to know more about exclusion from access to information due to a digital divide in access to broadband internet services.*	
**Question**	**Prompts**
Please describe the neighbourhood you lived in during COVID19 restrictions.	Facilities, amenities (e.g. access to supermarket/groceries, takeaways, food delivery services, local medical services) transport links, sense of community social cohesion (e.g. Teddy Bears in windows)
How did you contact local facilities and amenities, or stay in touch with people in your neighbourhood during COVID19 restrictions?	Landline, mobile phone, laptop/PC, email, video-call. Digital exclusion, mobile coverage, costs, satellite or fibre wifi, quality of connection. FaceBook community groups, telephone round robin. Over the fence.
Were you involved in any volunteering activities before COVID restrictions or receiving any help from volunteers or voluntary groups? If so, please tell me about these, and what happened once COVID restrictions started?	Local organisation, iwi, hapū, cultural group or church/faith-based organisation

**<u>PREAMBLE :</u> Now I want to ask you about the Government information that was issued COVID19 restrictions, how you accessed this information and whether it met your specific needs as a caregiver.**	
<u>PURPOSE:</u> *The* ***social structural and cultural*** *area of enquiry will explore whether information and resources developed during the pandemic addressed caregivers' needs, or whether there are unresolved issues that still need to be tackled in order for us to be prepared for future scenarios. For example, was guidance culturally safe and sensitive for Māori and Pacific caregivers? Were there suitable support systems for caregivers who lived at a distance from care recipients? And were generic resources suitable for caregivers supporting people living with dementia?*	
**Question**	**Prompts**
How did you find the information you needed to learn about COVID19 during the restrictions?	What did they do/who did they ask, online/telephone/in person. Culturally or linguistically safe, appropriate and accessible. Generic resources suitable for people living with dementia. Level of confidence in the information provided.
Was there any information about COVID19 itself or the alert levels that worked really well for you or was especially helpful?	
Was there any other information you think should have been provided during COVID19 restrictions, but wasn't?	
Before I conclude this interview is there anything else you would like to say about providing care during the COVID19 restrictions?	
