# Supplementary material for: Health equity and wellbeing among older people’s caregivers in New Zealand during COVID-19: Protocol for a qualitative study
Source: PLoS One. 2022 Jul 15;17(7):e0271114. doi: 10.1371/journal.pone.0271114 (PMC9286244; doi:10.1371/journal.pone.0271114)
Supplement: Attachment — Submitted filename: PONE-D-21-26434_reviewer.pdf [file pone.0271114.s005.pdf]

PLOS ONE

Health equity and wellbeing among older people's caregivers in New Zealand during COVID-19: Protocol for a qualitative study

--Manuscript Draft--

**Manuscript Number:**	PONE-D-21-26434
**Article Type:**	Study Protocol
**Full Title:**	Health equity and wellbeing among older people's caregivers in New Zealand during COVID-19: Protocol for a qualitative study
**Short Title:**	Health equity and wellbeing among older people's caregivers (NZ) during COVID-19: Protocol
**Corresponding Author:**	Vanessa Burholt, PhD<br>University of Auckland<br>Grafton, Auckland, Auckland NEW ZEALAND
**Keywords:**	gerontology; caregivers; health equity; Quality of Life; Wellbeing; indigenous people; marginalised populations; ethnic minority group; New Zealand; COVID-19; rural health; rural disadvantage; dementia; health services; Pacific peoples; Māori
**Abstract:**	Background<br>Knowledge of the challenges unpaid caregivers faced providing care to older people during the COVID-19 pandemic is limited. Challenges may be especially pronounced for those experiencing inequitable access to health and social care. This participatory action research study, located in New Zealand, has four main objectives, (i) ~~to undertake interviews with caregivers, and select caregivers' letters from an extant study, in order~~ to understand the challenges and rewards associated with caregiving to older care recipients during the COVID-19 pandemic restrictions; (ii) ~~to undertake interviews with organisations providing services to caregivers and older people, in order~~ to map and collate resources developed (or mobilised) during the pandemic; (iii) to ~~hold workshops and a co-design challenge event~~ to co-produce policy recommendations, identify useful caregiver resources and practices, prioritise unmet needs (challenges); and, (iv) to use project results in knowledge translation, in order to improve caregivers access to resources, and raise the profile and recognition of caregivers contribution to society.<br>Methods and analysis<br>Māori, Pacific (Tongan, Samoan and Niuean) and rural-dwelling caregivers to 30 older care-recipients, and 30 representatives from organisations supporting caregivers in New Zealand will be interviewed. Combining data from the interviews and caregivers letters (from an archive of older people's pandemic experiences), framework analysis will be used to examine the interrelated systems of the human ecological model and the impact on caregiving experiences during the pandemic. Through co-production with caregivers and community partners we will produce three short films describing caregivers' pandemic experiences; identify a suite of resources for caregivers to use in future events requiring self-isolation, and in everyday life; and generate ideas to address unresolved issues.
**Order of Authors:**	Vanessa Burholt, PhD
	Deborah Balmer
	Rosemary Frey
	Pare Meha
	John Parsons
	Mary Roberts
	Mary Louisa Simpson
	Janine Wiles
	Moema Gregorzewski
	Teuila Percival
	Rangimahora Reddy

Additional Information:	
**Question**	**Response**
**Financial Disclosure**<br><br>Enter a financial disclosure statement that describes the sources of funding for the work included in this submission. Review the submission guidelines for detailed requirements. View published research articles from *PLOS ONE* for specific examples.<br><br>This statement is required for submission and **will appear in the published article** if the submission is accepted. Please make sure it is accurate.<br><br>**Unfunded studies**<br>Enter: *The author(s) received no specific funding for this work.*<br><br>**Funded studies**<br>Enter a statement with the following details:<br>• Initials of the authors who received each award<br>• Grant numbers awarded to each author<br>• The full name of each funder<br>• URL of each funder website<br>• Have or will the sponsors or funders play any role in the study design, data collection (when applicable) and analysis, decision to publish, or preparation of the manuscript?<br>• **NO** - Include this sentence at the end of your statement: *The funders had and will not have a role in study design, data collection and analysis, decision to publish, or preparation of the manuscript.*<br>• **YES** - Specify the role(s) played.<br><br>* typeset	This work was supported by Health Research Council of New Zealand (https://www.hrc.govt.nz/) Project Grant 20/1380 to Vanessa Burholt. The funders have not and will not have a role in study design, data collection and analysis, decision to publish, or preparation of the manuscript.
**Competing Interests**<br><br>Use the instructions below to enter a competing interest statement for this submission. On behalf of all authors, disclose any competing interests that could be perceived to bias this work—acknowledging all financial support and any other relevant financial or non-financial competing interests.	The authors have declared that no competing interest exist.

<table>
<tr>
<td>This statement is required for submission and **will appear in the published article** if the submission is accepted. Please make sure it is accurate and that any funding sources listed in your Funding Information later in the submission form are also declared in your Financial Disclosure statement.

View published research articles from *PLOS ONE* for specific examples.

NO authors have competing interests

Enter: *The authors have declared that no competing interests exist.*

Authors with competing interests

Enter competing interest details beginning with this statement:

I have read the journal's policy and the authors of this manuscript have the following competing interests: [insert competing interests here]

* typeset</td>
<td></td>
</tr>
<tr>
<td>**Ethics Statement**

Enter an ethics statement for this submission. This statement is required if the study involved:

• Human participants
• Human specimens or tissue
• Vertebrate animals or cephalopods
• Vertebrate embryos or tissues
• Field research

Write "N/A" if the submission does not require an ethics statement.

General guidance is provided below. Consult the submission guidelines for detailed instructions. **Make sure that all information entered here is included in the**</td>
<td>Written ethical approval was obtained from the Auckland Health Research Ethics Committee (reference AH21966) on the 31 May 2021.</td>
</tr>
</table>

**Methods section of the manuscript.**<br><br>**Format for specific study types**<br><br>**Human Subject Research (involving human participants and/or tissue)**<br>• Give the name of the institutional review board or ethics committee that approved the study<br>• Include the approval number and/or a statement indicating approval of this research<br>• Indicate the form of consent obtained (written/oral) or the reason that consent was not obtained (e.g. the data were analyzed anonymously)<br><br>**Animal Research (involving vertebrate animals, embryos or tissues)**<br>• Provide the name of the Institutional Animal Care and Use Committee (IACUC) or other relevant ethics board that reviewed the study protocol, and indicate whether they approved this research or granted a formal waiver of ethical approval<br>• Include an approval number if one was obtained<br>• If the study involved *non-human primates*, add *additional details* about animal welfare and steps taken to ameliorate suffering<br>• If anesthesia, euthanasia, or any kind of animal sacrifice is part of the study, include briefly which substances and/or methods were applied<br><br>**Field Research**<br><br>Include the following details if this study involves the collection of plant, animal, or other materials from a natural setting:<br>• Field permit number<br>• Name of the institution or relevant body that granted permission	
**Data Availability**<br><br>Authors are required to make the data underlying their research findings fully available, without restriction.<br><br>If the manuscript reports pilot or preliminary results, the data underlying	N/A - the protocol does not report results

those results must be made available at the time of publication. A Data Availability Statement describing where the data can be found is required at submission. Your answers to this question constitute the Data Availability Statement and will be published in the article, if accepted. When legal or ethical requirements prohibit public data sharing, authors must indicate how researchers may obtain access to the data. See the PLOS Data Policy and FAQ for detailed information.<br><br>**Important:** Stating 'data available on request from the author' is not sufficient. If your data are only available upon request, select 'No' for the first question and explain your situation in the text box.<br><br>For protocols without pilot or preliminary data, authors are strongly encouraged to state how they plan to share research data from their study when it is completed or published.<br><br>Do the authors confirm that EITHER the protocol does not report results OR all data underlying the preliminary results will be made fully available without restriction?	
**Describe where the data may be found in full sentences. If you are copying our sample text, replace any instances of XXX with the appropriate details.**<br><br>• If **no pilot data are reported** in the manuscript, please enter the following: *No datasets were generated or analysed during the current study. All relevant data from this study will be made available upon study completion.*<br>• If the manuscript reports pilot data **held in a public repository**, include URLs, accession numbers or DOIs. If this information will only be available after acceptance, indicate this by ticking the	Data will not be shared publicly when the study is completed and published because of Māori (indigenous population) Data Sovereignty

box below. For example: *All XXX files are available from the XXX database (accession number(s) XXX, XXX.).*<br>• If the manuscript reports pilot data contained **within the manuscript and/or Supporting Information files**, enter the following:<br>*All relevant data are within the manuscript and its Supporting Information files.*<br>• If neither of these applies but you are able to provide **details of access elsewhere**, with or without limitations, please do so. For example:<br><br>*Data cannot be shared publicly because of [XXX]. Data are available from the XXX Institutional Data Access / Ethics Committee (contact via XXX) for researchers who meet the criteria for access to confidential data.*<br><br>*The data underlying the results presented in the study are available from (include the name of the third party and contact information or URL).*<br>• This text is appropriate if the data are owned by a third party and authors do not have permission to share the data.<br><br>For study protocols with data management or sharing plans, authors are encouraged to briefly describe in their Data Availability Statement how data they generate will be made accessible when the study is completed. For example:<br>• Deidentified research data will be made publicly available when the study is completed and published.<br><br>* typeset	
Additional data availability information:	

Manuscript Click here to access/download;Manuscript;Protocol V2.3.docx

Health equity and wellbeing among older people's caregivers in New Zealand during COVID-19: Protocol for a qualitative study

Vanessa Burholt[1,2,3,*], Deborah Balmer[1¶], Rosemary Frey[1¶], Pare Meha[5¶], John Parsons[1¶], Mary Roberts[6¶], Mary Louisa Simpson[7¶], Janine Wiles[2¶], Moema Gregorzewski[4¶], Teuila Percival[6#], Rangimahora Reddy[5#]

1. School of Nursing, Faculty of Medical and Health Sciences, University of Auckland, Grafton, Auckland, New Zealand

2. School of Population Health, Faculty of Medical and Health Sciences, University of Auckland, Grafton, Auckland, New Zealand

3. Centre for Innovative Ageing, College of Human and Health Sciences, Swansea University, Wales, United Kingdom

4. Centre for Arts and Social Transformation, Faculty of Education and Social Work, University of Auckland, Epsom, Auckland, New Zealand

5. Rauawaawa Kaumātua Charitable Trust, Hamilton, Waikato, New Zealand

6. Moana Research, Māngere, Auckland, New Zealand

7. Waikato Management School, University of Waikato, Hamilton, New Zealand

*Corresponding author:

Vanessa.burholt@auckland.ac.nz

VB is lead author

¶ These authors contributed equally to this article

# These authors contributed equally to the protocol that was submitted to the funders and their work has been used in this article

Abstract

Background

Knowledge of the challenges unpaid caregivers faced providing care to older people during the COVID-19 pandemic is limited. Challenges may be especially pronounced for those experiencing inequitable access to health and social care. This participatory action research study, located in New Zealand, has four main objectives, (i) to undertake interviews with caregivers, and select caregivers' letters from an extant study, in order to understand the challenges and rewards associated with caregiving to older care recipients during the COVID-19 pandemic restrictions; (ii) to undertake interviews with organisations providing services to caregivers and older people, in order to map and collate resources developed (or mobilised) during the pandemic; (iii) to hold workshops and a co-design challenge event to co-produce policy recommendations, identify useful caregiver resources and practices, prioritise unmet needs (challenges); and, (iv) to use project results in knowledge translation, in order to improve caregivers access to resources, and raise the profile and recognition of caregivers contribution to society.

Methods and analysis

Māori, Pacific (Tongan, Samoan and Niuean) and rural-dwelling caregivers to 30 older care-recipients, and 30 representatives from organisations supporting caregivers in New Zealand will be interviewed. Combining data from the interviews and caregivers letters (from an archive of older people’s pandemic experiences), framework analysis will be used to examine the interrelated systems of the human ecological model and the impact on caregiving experiences during the pandemic. Through co-production with caregivers and community partners we will produce three short films describing caregivers’ pandemic experiences; identify a suite of resources for caregivers to use in future events requiring self-isolation, and in everyday life; and generate ideas to address unresolved issues.

Introduction

In 2019, a cluster of people with pneumonia of unknown aetiology were identified in Wuhan, China [1] and by January 30 the World Health Organization declared the coronavirus disease 2019 (COVID-19) outbreak as a Public Health Emergency of International Concern [2]. In response to the COVID-19 pandemic, in April 2020 the Government of New Zealand (NZ) introduced a four-level alert system. At Alert Level 2 and higher, the Government recommended that people at higher risk of mortality from contracting COVID-19 should stay at home. Those at ‘high risk’ included older people (70+ years, or younger for Māori [the indigenous people of NZ] and Pacific peoples) and those with underlying health conditions [3] which are prevalent in the older population. During the pandemic, some older people in NZ will have spent more than 100 days confined to their home. In many cases, unpaid kin and non-kin caregivers have supported older people by providing care and assistance with activities of daily living and home health care. In this study we use the term ‘unpaid caregiver’ to describe someone who provides care and support primarily because of a personal kin or non-kin relationship with the care-recipient. We do not use the phrases ‘informal care’ ‘care partner’ or ‘carer’. The former contributes to the invisibility of unpaid caregivers in society as it conveys the impression that roles are unofficial (i.e. not defined by a salary, title or duties) and ‘not essential’ [4], while the latter do not clearly distinguish unpaid caregivers from paid care providers.

Caregiving in the community is often under-appreciated [5]. However, global health and social care systems are so reliant on unpaid caregivers that they constitute an essential workforce [6, 7]. In the international response to control COVID-19 transmission, unpaid caregiving has, and continues to be, the ‘backbone’ of health and social care provision in the community. However, the voices and stories of family carers have been invisible, and our knowledge of the successes, rewards, and challenges faced by those providing care to older

people is limited. To date, articles assessing the situation of caregivers during COVID-19 have tended to focus on individual psychosocial outcomes such as depression [8], stress [9], burden [10, 11], suicidal tendencies [12], substance abuse [12], and psychological distress [13].

This research intends to raise the profile of unpaid caregivers providing essential support to older people, including those living with dementia, by recognising their significant social role during the COVID-19 pandemic in NZ. The research will generate new knowledge by exploring how family caregiving roles, access and use of resources changed. It will identify positive aspects of caregiving alongside unmet needs and challenges. The research will put knowledge into action by collating and co-designing resources for family caregivers to support their care of older people. Pooling knowledge about caregiving during COVID-19 with international study teams (e.g. Southwest Health Equity Research Collaborative in Northern Arizona University), regarding caregiving in indigenous and minority populations, in the context of rurality and caregiving for people living with dementia, will facilitate cross-national knowledge transfer. Mapping responses in different countries aimed at supporting caregivers during the COVID-19 restrictions will help us identify policy, programmes or resources with the potential to improve national public health strategies and preparedness for future emergency responses to achieve the best possible outcomes for caregivers.

Unpaid caregiving to older care recipients in NZ pre-COVID-19: The scale of the issue

Estimates suggest that 480,000 people (in a population of approximately 5 million) provide regular care for someone with an illness or disability [14]. This figure underrepresents spouses, Māori and Pacific caregivers [15].

Caregivers in NZ are most likely to be between 50-54 years, and provide on average 30 hours or more per week of care [16] for 8 years and 10 months [17]. However, significant numbers of younger people, older people, and adult children provide care to older people [18], for example, 34% of caregivers who provide care for household members with illness/disabilities are aged 55+ years [19]. Care may be provided to an older person by a number of caregivers [20] and 65% of caregivers are simultaneously in paid employment [14]. Caregivers' ethnicity reflects the population in general, with Māori and European New Zealanders most likely to report providing care. European New Zealander caregivers are more likely to report providing support to someone living in a separate household, than caregivers of other ethnicities who are more likely to report co-residence [15].

Caregiving for older people living with dementia is a major public health issue for societies worldwide. It is estimated that there are presently 70,000 people living with dementia in NZ, and the number is predicted to increase to 170,212 by 2050 [21]. In 2020, almost 87% of those with dementia in New Zealand were of European ethnicity, with Maori and Pacific people accounting for 6.2% and 2.8% of the population of people living with dementia respectively. Dementia is the largest single contributor to disability and need for care among older adults [22]. Globally, the vast majority of support and care for people living with dementia is provided through unpaid kin/non-kin networks (including spouses, adult children, children-in-law, and friends) [23].

In 2016, there were approximately 40,000 carers providing 45 million hours of care annually to people living with dementia in NZ, with an estimated cost of NZ$68.6 million [21]. Under normal circumstances, the provision of care and support to a people living with dementia in the community can result in significant physical, emotional and economic strain for caregivers [24-30]. While we expect some features of caregiving to be similar regardless of the functional or cognitive characteristics of the person being cared for during the COVID-

19 pandemic, we also anticipate that caregivers to people living with dementia faced some unique challenges (e.g. disruption of basic routines that promote mental health, and the unavailability of memory clinics and respite care [31]).

Multilevel influences on caregiving pre COVID-19: The breadth of the issue

Pre-COVID-19 pandemic research identified several layers of influence on the caregiving experience. Within a human ecological systems framework, outcomes for caregivers and care recipients are influenced by social structural (public policy) and cultural norms, community, institutional/organisational factors, interpersonal processes and intrapersonal factors and the interaction between these factors (Fig 1).

Fig 1. Human ecological model comprising inter-related systems that influence caregiving

Social structural and cultural influences on caregiving

The NZ healthcare system depends heavily on caregivers to provide support for older people [32]. Although government policies draw attention to the need to recognise the important role of family caregivers [33, 34] there remains little acknowledgment of their critical contributions to society [35-37]. National and local policies are responsible for eligibility and allocation of resources to care-givers/recipients, but bureaucratic obstacles [38] and difficulties with navigating health and social care systems present barriers to accessing services [39-41]. Some policies suggest that apps, online resources and social media may facilitate caregivers' connections with service and information, especially in isolated

communities [33, 42], while others acknowledge that caregivers may need navigational help to receive the support and information they need [34]. The current Carers' Strategy Action Plan 2019–2023, *Mahi Aroha*, states that caregivers should be supported to continue in employment and to fully participate in a social life while providing support [34]. Despite these policy commitments, there is evidence of a strong direct relationship between caregivers' increasing sense of burden and discontinuation in their caring role [43].

In addition to public policy, there are cultural influences on caregiving. The terminology caregiver may not resonate with everyone and there are cultural differences in usage [44]. Obligations to provide care that are based on normative expectations of family solidarity (prevalent in Pacific and Māori communities) may not be referred to as 'caregiving', and is an important reason why caregivers do not seek out services [45].

For Pacific peoples, older people are revered and caring for them is a prioritised service to one's family [46, 47]. Samoan people, for example, believe that caring for elders is an obligation and in return children will gain blessings (manuia).

For Māori, family (whānau) care of older people (kaumātua) is founded on commitment to reciprocal relationships and obligations to help [48] where older people are "carriers of culture, anchors for families, models for lifestyle, bridges to the future, guardians of heritage, and role models for younger generations" [49]. Family care is perceived as "normal" [50-52] and critical to the wellbeing of older people in the context of significant ageing and health inequities for Māori [53-57]. In caring for older people with dementia, family are crucial in promoting a healthy sprit or soul (wairua) [51]. The collective obligations of the family are informed by cultural concepts such as love (aroha), showing respect, generosity and care for others (manaakitanga), genealogy (whakapapa) and relationships building through shared experiences and working together which provides a sense of belonging (whanaungatanga) [51]. Māori family caregiving sits at the heart of the

wellbeing approach to care work (mahi aroha) integrating cultural identity, sustainability of the Māori world view (Te Ao Māori), social connections, knowledge and skills, human resource potential, and income [34].

Very little is known about the support needs of Māori family-based care networks, or Pacific care networks although caregivers have multiple demands on their time [58-60]. Pacific [61, 62] and Māori caregivers [51] are among those most in need of support, but are less likely to be aware of available services [45] and more likely to have lower levels of health literacy than European New Zealanders [63-65]. The additional challenge of English as a second language creates barriers in accessing health care services [61]. Both Māori and Pacific peoples have reported discrimination in health care services [66]. It has been suggested that health care providers need to work on their cultural competency, communicating information that resonates with the values and contexts of Māori and Pacific people, and developing appropriate outreach mechanisms to identify and connect with caregivers from different cultural groups [67-69].

Community/neighbourhood influences on caregiving

In NZ, caregivers face significant challenges according to their geographic location or area deprivation [70]. The remoteness of communities affects access to health, social care and other essential (e.g. groceries) or community services [42, 71]. People living in remote rural areas are less likely to have a landline or cell phone coverage [42, 72]. Furthermore, area disadvantage may impact negatively on health outcomes for older caregivers [73].

Neighbourhoods have the potential to positively shape health, quality of life, and well-being for caregivers through the provision of local services, a sense of attachment to place [74, 75] and social interactions in 'third spaces' (e.g. coffee shops, parks and libraries) [76, 77]. In places with fewer opportunities for community participation and lower levels of

social capital, caregivers of people living with dementia are more likely to report higher levels of isolation and find it harder to manage the demands of care [78].

In rural communities, there may be increased emphasis on family support, because of reduced options for alternative sources of support [79]. However, rural communities may be subject to ongoing population change and instability, and sources of formal or informal support may be stretched thinly [80-82]. Additionally, there may be an increased risk of abusive caregiver-recipient relationships, as there can be limited contact with people who could identify abuse or offer support in rural communities [79].

Organisational influences on caregiving

Unpaid caregivers perform an essential function as 'care-coordinators' linking older care-recipients to health care services, including primary health care and general practice. Caregivers attempt to meet gaps in formal health and social services for older adults [6, 83-89], advocating for care recipients,[41, 83, 90-92], facilitating access to, and explaining health information [84, 93]. High-quality care at home relies upon strong relationships between health professionals and caregivers, good communication [60, 94, 95], and collaborative working [33, 96]. In NZ, services and support for caregivers is provided through primary care, District Health Board funded community services (e.g. Needs Assessment Support Coordination) and non-governmental organisations [41, 97] and the Supported Living Payment. Despite some recent effective cross-sectoral coordination [41, 59, 60, 86-88] the uptake of caregiver support is low, indicating a lack of flexibility [41, 98], inadequate provision [41], lack of knowledge about services [99], and a disconnection between what is offered and what is needed [100]. Unmet needs for formal services places increased demands on caregivers [101] and organisations may provide services that are

neither relevant to caregivers needs and consistent with their wishes [38], nor socially, culturally or linguistically appropriate and accessible.

For working caregivers, the organisational characteristics of employers are also important. Juggling work and caregiving roles can impact negatively on health and well-being, especially where employers do not have carer support strategies or have inflexible working policies [102, 103]. The Employment Relations Flexible Working Arrangement Act 2007/2008 allows employees to request flexible working arrangements, however, fewer than one-third of workers are aware of this and many use annual or sick leave to provide care [104]. Furthermore, many caregivers reduce working hours or exit the workforce in order to provide care to an older person [102, 105], often negatively influencing their financial resources [106].

Family, social networks, interpersonal relationships influence on caregiving

Unpaid care-networks of older people may comprise partners, family networks, or mixed networks (care from children, other family members and professionals) [107-111] and the quality and extent of support are critical to caregivers' experiences. Particular caregiver-recipient relationships may produce different outcomes, for example, spousal caregivers have worse mental health outcomes than adult-child caregivers [112]. Furthermore, not all caregiver-recipient relationships are positive: conflict, tension and negative interactions are salient [113, 114]. Kin-networks may be disrupted by divorce and remarriage which may alter intergenerational bonds and responsibilities. Negative interactions within stepfamilies or discordant coordination of care within families can lead to greater levels of depression and caregiver burden [113, 115]. The risk of abusive relationships increases when individuals, families are isolated, or when caregivers do not receive appropriate support (e.g. coping with particular challenges associated with dementia) [15].

Individual characteristics and resources influence on caregiving

A large body of global research emphasises negative impacts of caregiving on physical, psychosocial, emotional, social and financial circumstances, otherwise known as 'caregiver burden' [116]. In particular, the risks for poor mental and physical health associated with caregiving have been highlighted [117-119], especially for caregivers of people living with dementia [24-29]. In NZ, caregivers are more likely to experience poor health [106] depression and anxiety than non-caregivers [120]. Research suggests that female caregivers and Māori caregivers have the poorest mental health [17].

Elsewhere, research has demonstrated that caregiving elicits a high economic burden [18]. However, little is known about the costs incurred by family caregivers in NZ [20]. Caregivers may experience out-of-pocket expenditure on services (e.g. personal care for care recipient or respite care), goods (e.g. bedding, mobility aids and incontinence products), home adaptations to improve accessibility, increased heating, laundry and transportation fees [121], but may also incur a loss of income associated with changes to employment [18]. Caregivers in NZ have lower economic living standards compared to non-carers [106]. An inadequate income increases the risk of poor health and depressive symptoms for caregivers [122], and can restrict access to private care services [38].

As identified above, much of the academic literature on caregiving to older people focuses on negative outcomes, implying that it is financially physically and psychologically harmful [117] . This has been referred to as the 'penalties of caring' [44]. However, it is also important to recognise positive aspects of caregiving that are often ignored or not assessed. Being able to provide care for a loved one is often highly valued [77, 123]. Emphasising positive aspects of the role can improve the sense of self-efficacy, feelings of accomplishment, the quality of the caregiver/care-recipient relationship and reinforce caregiver wellbeing [124].

Unpaid caregiving to older care recipients during COVID-19: Our knowledge of the issue

Knowledge of the impact of COVID-19 on caregivers is emerging. For example, a survey by Carers NZ/NZ Carers Alliance provided an insight into the experiences of 676 caregivers during the COVID-19 pandemic [125]. The survey highlighted several issues including reduced access to services and essential supplies, concerns about paid care staff coming into contact with the care recipient [126], increased exhaustion and stress, and financial pressures. Although the survey provides an indication of some challenges, we are unable to distinguish between the concerns of caregivers providing support to older people or people living with dementia from others, or whether caregivers from different cultures/ethnicities, social or geographic locations experienced particular inequities in health and care provision. From our understanding of the factors that impacted on caregivers before the global pandemic, we can postulate that some of these will have been amplified during COVID-19 as the related measures (socially distancing/isolating) are likely to have been an additional contextual stressor [127].

At the policy level, while general announcements and updates were praised for keeping the population of NZ informed about key developments during the pandemic [125] to date, no research has assessed whether the information and resources addressed caregivers' needs, or whether there are unresolved issues that still need to be tackled in order for us to be prepared for future scenarios. For example, was guidance culturally safe and sensitive for Māori and Pacific caregivers [128]? Were there suitable support systems for caregivers who lived at a distance from care recipients? And were generic resources suitable for caregivers supporting people living with dementia?

At the organisational level we need to understand whether clinical home care service guidelines need updating in light of caregivers experiences during the pandemic. We also

need to understand how employers supported working carers, and how this impacted on the caregiving experience.

At the community level, framing unpaid caregiving through families in terms of reciprocity, obligation, solidarity and love [129] is in danger of glossing over issues of social justice and inequity at the community level. Some communities could be described as impoverished care environments and may be least equipped to meet a such a social care agenda [130]. We need to identify inequities in health and social care support, and access to others services for caregivers in disadvantaged communities or living at a distance from ‘resource centres’ [131]. We also need to know more about exclusion from access to information due to a digital divide in access to broadband internet services [132] and whether caregivers living in communities that experienced inequities before lockdown have been pushed further to the margins.

At the interpersonal level, many caregivers rely on the stability and connection of their family and kinship systems. We do not know the extent to which family and caregiving networks were hampered or enabled by social distancing requirements during COVID-19. The caregiver-care recipient relationship may have also been strained due to increases in caregiving tasks, hyper-vigilance, and less respite.

At the individual level, in addition to the greater impact on personal resources, COVID-19 probably altered the daily life of employed caregivers [126, 133, 134]. In addition, some caregivers may have faced additional child-care pressures exacerbated by school and nursery closures. Research in the United Kingdom suggests that caregivers sought to reduce their own risk of infection, to protect older care recipients [126]. Consequently, caregivers employed in ‘essential services’ may have felt conflicted about undertaking work duties that could potentially put an older care-recipient at risk of infection. We need to understand the situation of caregivers (for older people with/out dementia), in terms of

burden, physical and mental wellbeing, burnout [128], sleeplessness [135], social isolation and loneliness [136].

A critical human ecological framework to examine caregiving during COVID-19

Globally, unpaid caregivers are a crucial human resource that improve health-care capacity especially in areas with inequitable/sub-optimal access to health and social care. A critical human ecological approach [137] provides a meta-framework for challenging inequities and will be used to question taken-for-granted assumptions about caregivers during the COVID-19 pandemic. These assumptions included the continuing capacity of caregivers to provide care, their psychological-preparedness to provide support, their functional capacity to cope with demanding and physical caregiving tasks without respite [128], and their financial capacity to bear the brunt of additional caregiving costs. Additionally, there were implicit assumptions that caregiver-recipient relationships would be mainly co-resident dyads with little attention paid to the implication of social restrictions for non-co-resident caregivers, or caregiving that was distributed throughout families.

This research will examine the intersectionality of structural inequities, focusing on subgroups of caregivers who may face particular challenges, or may be excluded from existing policy [138], that is, those living in remote, rural disadvantaged areas [139], and Māori and Pacific (Tongan, Samoan and Niuean) caregivers. Caregiving will be conceptualised as an emergent product of a system, in which individual, community, environment and macro-structures (e.g. governmental policies, culture, values and normative beliefs) are inextricably connected [140].

While this research will highlight health and social inequities, the COVID-19 pandemic also provides a unique opportunity to maximise learning from any positive ways in which caregiving was disrupted. For example, opportunities to work at home [126], or to receive remote medical consultations (telemedicine), may have provided benefits that could or should be extended into life beyond the pandemic to improve quality of life. Taking a citizen-centred approach, the goal of this research is to provide the opportunity for caregivers in NZ to have a voice in shaping recommendations for future health and social policy, and services [141]. It will contribute to the development of solutions that are culturally responsive and relevant to NZ. Our international collaborations will provide the opportunity to identify experiences that were common to caregivers during the pandemic and point to vulnerabilities and areas that may require national and international reform to reduce the risk of future public health crises impacting on caregivers.

Objectives

The objectives of this study are:

- To undertake interviews with caregivers, and select caregivers' letters from an extant archive, in order to understand the challenges and rewards associated with caregiving to older care recipients during the COVID-19 pandemic restrictions.
- To undertake interviews with organisations providing services to caregivers and older people, in order to map and collate resources developed (or mobilised) during the pandemic.
- To hold workshops and a co-design challenge event to co-produce policy recommendations, identify useful caregiver resources and practices, prioritise unmet needs (challenges).

- To use project results in knowledge translation, in order to improve caregivers access to resources, and raise the profile and recognition of caregivers contribution to society.

Research questions

The research will address the following questions:

1. What were the experiences of Māori, Pacific (Tongan, Samoan and Niuean) and rural-dwelling caregivers (providing care to older people) in NZ during the COVID-19 pandemic?
2. What were the similarities and differences between the experiences of caregivers for people living with dementia compared to caregivers providing care for older people with other health and disability needs?
3. How did the interrelated systems of the human ecological model impact on caregiving experiences during the COVID-19 pandemic?
4. What can we learn from the experiences of caregivers and organisations supporting them, to prepare for other similar (emergency health) situations, or to transfer to new ways of working in everyday life?

Methods

This study uses take a participatory action research approach [142, 143]. Phases 1-2 (concurrent qualitative studies) will underpin co-design activities in Phase 3 (transformative sequential phase). The co-production of knowledge is often referred to as integrated knowledge translation which describes an ongoing relationship between researchers, community groups and policy-makers for the purpose of engaging in a mutually beneficial research to support decision-making [144, 145]. Using participatory action research,

integrated knowledge translation [146] will be achieved through a reflective cycle, whereby community partners collect and analyse data, and along with caregivers, identify what actions should follow.

Four main elements underpin our co-created research: (i) collaboration and equitable involvement in research; (ii) solidarity and capacity-building; (iii) empowerment and action for systems change; and (iv) sustainability [142]. Accordingly, all partners have actively contributed to the proposal, [142] in order to deliver customised research to improve local practice [147].

Phase 1: Experiences of caregivers

Sample and setting

Two samples will be used in Phase 1 of the study, extant data and new primary data.

1. Extant data for caregivers extracted from a study entitled *Social connectedness among older people during Covid-19* (Auckland Medical Research Foundation; Principal Investigator Professor Merryn Gott). This study requested letters from people age ≥ 70 years living in NZ describing their experiences of COVID-19. Data collected between May 2020 and May 2021 comprises >650 responses. We will select letters sent by older caregivers from the main dataset by searching files for key words ('care' 'support' and synonyms).
2. A new cross-sectional convenience sample of caregivers providing support to 15 older people living with dementia, and 15 without dementia (i.e. caregivers to five older people living with dementia and five without in each group: Māori, Pacific and rural). Established and trusted relationships are critical to conducting community research [148]. Participants will be volunteers identified through our collaborating community

group networks who live and work within the communities.

Study design

In a cross-sectional qualitative study, caregivers who provide care or support to an older person, within the three groups/settings will be invited to take part in face-to-face guided interviews in their own homes, or in a setting of their choice. Individual, joint, or family-based interviews will be offered to participants. Consequently, the number of caregivers interviewed (providing care to 30 care-recipients) is likely to be greater than 30. Face-to-face interviews are preferred by Māori caregivers [121] and caregivers with mobility, hearing or vision difficulties who may encounter difficulties with written responses or telephone interviews [149, 150]. However, if the Alert Levels associated with the COVID-19 pandemic are in place and we are unable to interview face-to-face, we will adopt a contingency plan and conduct interviews by Zoom/Skype/ telephone [151].

A short questionnaire will be used to establish the demographic characteristics of the caregiver(s) (S1 Appendix, Demographic Questions: Caregivers). An interview guide will be aligned to the 'levels' of the human ecological model and will focus on the individual, interpersonal relationships, organisational issues, community and public policy and culture (S2 Appendix, Semi-Structured Interview Guide for Individual Caregivers). The first area of enquiry will focus on the individual and is structured to establish changes in caregiving during COVID-19 restrictions and positive and negative outcomes on personal resources and wellbeing. This section will also explore whether there were challenges balancing caregiving and additional roles (e.g. mother, employee or employer) during COVID-19 restrictions. The second section will explore family, social networks, interpersonal relationship in relation to caregiving. The third section, the organisational area of enquiry, will establish whether clinical home care service guidelines need updating in light of experiences during the

pandemic. The topic guide will cover issues relations to encounters with home care providers during COVID-19, use of telecare and telehealth, alongside issues concerning the availability of resources (e.g. PPE, respite, day care), and the impact on caregivers. The fourth section, focusing on the community/neighbourhood area of enquiry, will identify inequities in access to services (including broadband and internet services) for caregivers in disadvantaged communities and for those living at a distance from resource centres. The fifth section will address the social structural and cultural area of enquiry, and will explore whether information and resources developed during the pandemic addressed caregivers' needs, or whether there are unresolved issues that still need to be tackled in order to be fully prepare for future scenarios. A modified interview guide will be used for group interviews with two or more caregivers: some questions will be directed at the main caregiver, while others will be asked of each participating caregiver (S3 Appendix, Semi-Structured Interview Guide for Groups [>1 Caregiver]).

Community Researchers working with Māori and Pacific caregivers will interview participants in Te Reo Māori (the Māori language), Samoan, Tongan, Niuean or English according to the caregivers' preferences. Face-to-face interviews will be informed by culturally aligned research ethics and processes. All participants will be offered NZ$50 voucher for their involvement in the research [152-154].

Analysis

A thematic analysis of secondary and primary cross-sectional qualitative data will be undertaken. Transcripts will be anonymised [155], imported and analysed using QSR NVivo Version 12. Drawing on social phenomenology [156] analysis will utilise both inductive and deductive thematic analysis to interpret the raw data within a framework analysis [157].

Familiarisation, conceptual and cultural understanding of the interviews will be clarified during team meetings. A first version of the coding index will comprise top level headings for themes heavily rooted in a priori issues (i.e. human ecological model). Two interviews from each group will be independently coded by the researchers during which categories (sub themes) will be identified and refined. The resulting index (comprising top level heading and clustered sub-themes) will be systematically applied to the text. Data will be charted into a framework to provide decontextualised descriptive accounts in relation to the different levels of the model and experiences during COVID-19 for each care-recipient (from the perspective of one or more caregiver). Schematic diagrams will be developed and used alongside the charted material to capture commonalities of experiences across caregivers [158, 159] and at each level of the model (e.g. identifying key issues relating to community disadvantage). Chronological explanatory summaries capturing the complexity of experiences will be created for each participant describing the interrelationship between levels of the model [160].

A Māori Expert Advisory Group and the collaborating Pacific Research Group will help guide interpretation of Māori and Pacific data analysis. Researchers will meet regularly to discuss different ways of interpreting the research phenomenon, differences between groups, and the persuasiveness of the analysis [161].

Phase 2: Organisational support and resources for caregivers

Sample and setting

In a cross-sectional qualitative study, a purposive sample of 30 national and local organisations across NZ (including Māori and Pacific organisations) who provided support to caregivers during COVID-19 will be selected through collaborating community

organisations.

Study design

CEOs/Directors of the service provider organisations will be contacted by phone/email to describe the purpose of the study. An appointment for a 30-45 minute telephone/Zoom interview will be made with the CEO/Directors or with a nominated member of the organisation. Guided interviews will follow a similar format to caregivers' interviews. The organisational representative will be asked for their perception of caregivers' successes and challenges during COVID-19. They will be asked to describe the organisational response to caregiver needs and asked to describe any resources/services that they developed specifically to address these (S4 Appendix, Semi-Structured Interview Guide for Service Providers).

Analysis

Telephone/Zoom interviews will not be recorded or transcribed verbatim. As these interviews do not require a particular closeness between researchers and the interviewees, we will use a reflexive, iterative process of data management comprising concurrent note-taking; reflective journaling immediately post-interview; summarising the description of resources, and undertaking preliminary content analysis identifying emerging themes [162].

The interviewee will be sent a (i) description of resources deployed/developed by the organisation and (ii) the major themes elicited in relation to the perception of caregivers (un)met needs. The participant will be asked to verify and/or amend the summaries to ensure stakeholder engagement and 'member checking' [163, 164]. The themes will be added to the

framework analysis (from Phase 1). The descriptions of resources will summarised into ‘plain’ language. For informational resources hard copies or URLs will be obtained.

Phase 3: Co-produced resources, recommendations and priorities for caregivers

Sample and setting

Caregivers who highlight an interest during Phase 1 will be eligible to participate in any of the co-creation workshops. We will organise six co-creation workshops (two in each setting) to assess the adequacy of collated caregiving resources and prioritisation of unresolved challenges. We anticipate a maximum of five participants in each of these workshops. We will convene one creative co-design challenge event [165]. Caregivers from Phase 1 that express an interest in telling their stories on film will be invited to take part in digital storytelling.

Study design

We will convene six workshops (two each with Māori, Pacific and rural caregivers) for ideation concerning appropriate recommendations and resource materials/models to support family caregivers. Each workshop will last about 2-3 hours. They will be conducted by researchers skilled in hosting co-production events, and will use cultural ways of working. The first event will cover the following issues:

- A summary of the key challenges faced by caregivers in each setting identified in Phase 1
- A summary of the resources deployed by organisations to meet caregivers’ needs identified in Phase 2

- Discussion around whether resources met caregivers' key challenges during COVID-19
- Identifying good practice for future health disasters (pandemics) and emergency situations
- Identification of good practice that could be applied to everyday life (extended to beyond pandemic preparedness) to support caregivers

At the second event, the participants will discuss insights, ideas for new practices, services, policy recommendations: with particular attention given to identified unmet needs (identified at the first workshop event) for information, practical health and social care support, health literacy, and psychosocial help. Ideas generated for new resources would be prioritised by each group to ensure successful application of study findings.

In order to address any unresolved prioritised issues, a creative co-design challenge event would bring together stakeholders (e.g. caregivers from the workshops, policymakers, service providers, designers, technology companies) to address the challenges [165]. During the creative co-design challenge event, small teams would co-produce a brief and implementation plan to move each challenge forward beyond the project funding period. Results will inform the design of practice, policy and future collaborative studies to address areas of high priority as indicated by the caregivers.

Three short films (one each for Māori, Pacific and rural caregivers) would be co-created to communicate caregiver 'stories'. An experienced researcher and a filmmaker will work with three individuals or groups (e.g. a group of family caregivers) to construct and share caregivers' own authentic narratives. The caregivers' interview transcripts will be used as an aide-mémoire to create 'storyboards' which will serve as a visual layout for story construction. Each short film will comprise 5-6 minutes of visual narrative creating compelling accounts of caregiving experiences [166, 167].

Ethics

Ethical approval was obtained from the Auckland Health Research Ethics Committee (reference AH21966) on the 31 May 2021.

Results

Data collection (Phase 1 and 2) commenced in June 2021 and will be completed by September 2021. Analysis of data will feed into Phase 3 to develop caregiver resources, digital stories, recommendations for organisational good practice, policy and coordination.

An integrated knowledge transfer strategy [144-146] will maximise the impact of outputs from the research within and beyond the grant lifecycle. Co-production and working with community-based partners and caregivers will result in realistic real-world application and will facilitate a pathway to impact through implementation of recommendations in primary care and maximising caregiver access to support resources.

Discussion

The need for this research was identified by our community partner groups. Subsequently, community groups and academics have risen to the challenge by jointly and collaboratively developing a proposal to identify and address the needs of specific sub-groups of caregivers who are likely to have been particularly affected by sub-optimal and inequitable access to advice, health and social care support and resources – that is Māori, Pacific (Tongan, Samoan and Niuean) and rural-dwelling caregivers. From the outset, the team has taken a participatory action research approach to project design which will continue throughout implementation to ensure translation of social research into public health benefit for family caregivers to older people (≈480,000).

We anticipate that the research will have some social, economic and cultural benefits. The research will improve caregivers' access to support resources, caregiving safety, effectiveness and cultural appropriateness during future emergency health situations. We will also identify good practice and resources developed during the pandemic that could be rolled out to positively impact on caregivers' everyday life. Furthermore, the research is anticipated to improve the responsiveness of health and social services to meet caregivers' needs, especially in populations that are vulnerable to exclusion, health and social inequity.

At the end of the study, resources will provide practical solutions for challenges associated with caregiving during and beyond the pandemic, especially for Māori, Pacific and caregivers living in remote, rural or disadvantaged areas. As shortfalls in support impact on caregiver burden, physical and cognitive health, quality of life, and other psychosocial outcomes (e.g. loneliness), we anticipate providing lasting societal benefits, potentially delaying institutionalisation of older care recipients, and reducing health and social care costs. The social, economic and cultural benefits will be achieved through the collation and distribution of best practice and support resources, recommendations for policy (especially the National Health Emergency Plan [168], in terms of readiness, recovery and response), and identifying unresolved challenges to be addressed beyond the funded duration of the project. Examining caregivers' experiences in more than one location with international partners will help to identify good practice, and perhaps clarify the drivers of suboptimal support, while deepening our knowledge about needs and expectations of caregivers. In turn, this could improve the quality of care, support and services, by broadening and strengthening evidence for change beyond the confines of a single nation.

References

1. Bogoch II, Watts A, Thomas-Bachli A, Huber C, Kraemer MUG, Khan K. Pneumonia of unknown aetiology in Wuhan, China: potential for international spread via commercial air travel. J Travel Med. 2020;27(2). doi: 10.1093/jtm/taaa008. PubMed Central PMCID: PMC7107534.
2. Mohile S, Dumontier C, Mian H, Loh KP, Williams GR, Wildes TM, et al. Perspectives from the Cancer and Aging Research Group: caring for the vulnerable older patient with cancer and their caregivers during the COVID-19 crisis in the United States. J Geriat Onc. 2020;11(5):753-60. doi: 10.1016/j.jgo.2020.04.010. PubMed Central PMCID: PMC7172832.
3. Onder G, Rezza G, Brusaferro S. Case-fatality rate and characteristics of patients dying in relation to COVID-19 in Italy. JAMA. 2020;323(18):1775-6. Epub 2020/03/24. doi: 10.1001/jama.2020.4683. PubMed PMID: 32203977.
4. Stall NM, Campbell A, Reddy M, Rochon PA. Words Matter: The language of family caregiving. J Am Geriatr Soc. 2019;67(10):2008-10. doi: 10.1111/jgs.15988. PubMed PMID: 31120551.
5. Archer J, Reiboldt W, Claver M, Fay J. Caregiving in quarantine: evaluating the impact of the Covid-19 Pandemic on Adult Child Informal Caregivers of a Parent. Gerontol and Geriatr Med. 2021;7:2333721421990150. Epub 2021/02/04. doi: 10.1177/2333721421990150. PubMed PMID: 33532515; PubMed Central PMCID: PMC7841668.
6. Bookman A, Harrington M. Family caregivers: a shadow workforce in the geriatric health care system? J Health Polit Policy Law. 2007;32(6):1005-41. doi: 10.1215/03616878-2007-040. PubMed Central PMCID: PMC18000158.
7. Lorenz-Dant K, Comas-Herrera A. The impacts of COVID-19 on unpaid carers of

adults with long-term care needs and measures to address these impacts: a rapid review of evidence up to November 2020. Journal of Long-term Care. 2021:124-53. doi: 10.31389/jltc.76.

8. Gallagher S, Wetherell MA. Risk of depression in family caregivers: unintended consequence of COVID-19. BJPsych Open. 2020;6(6):e119. Epub 2020/10/13. doi: 10.1192/bjo.2020.99. PubMed PMID: 33040759; PubMed Central PMCID: PMC7550870.

9. Cohen G, Russo MJ, Campos JA, Allegri RF. Living with dementia: increased level of caregiver stress in times of COVID-19. Int Psychogeriat. 2020;32(11):1377-81. Epub 2020/07/30. doi: 10.1017/S1041610220001593. PubMed PMID: 32729446.

10. Cohen SA, Kunicki ZJ, Drohan MM, Greaney ML. Exploring changes in caregiver burden and caregiving intensity due to COVID-19. Gerontol Geriatr Med. 2021;7:2333721421999279. doi: 10.1177/2333721421999279.

11. Cohen SA, Kunicki ZJ, Nash CC, Drohan MM, Greaney ML. Rural-urban differences in caregiver burden due to the COVID-19 pandemic among a national sample of informal caregivers. Gerontol Geriatr Med. 2021;7:23337214211025124. doi: 10.1177/23337214211025124.

12. Czeisler MÉ, Lane RI, Petrosky E, Wiley JF, Christensen A, Njai R, et al. Mental health, substance use, and suicidal ideation during the COVID-19 pandemic — United States, June 24–30, 2020. Washington DC: Centers for Disease Control and Prevention, 2020.

13. Park SS. Caregivers’ mental health and somatic symptoms during COVID-19. J Gerontol B Psuchol Sci Soc Sci. 2021;76(4):e235-e40. doi: 10.1093/geronb/gbaa121. PubMed PMID: 32738144; PubMed Central PMCID: PMC7454918.

14. Alpass F, Keeling S, Pond R. The New Zealand Longitudinal Study of Aging: summary

report. Caregiving. Palmerston North: Massey University, 2014.

15. Kia Piki Ake Welfare Expert Advisory Group. Current state: carers of people with health conditions or disabilities. Wellington: New Zealand Government, 2019.

16. Grimmond D. The economic value and impacts of informal care in New Zealand. Report prepared for Carers New Zealand and the NZ Carers Alliance. Wellington: Infometrics, 2014.

17. Alpass F, Szabo A, Allen J, Stephens C. Health effects of informal caring in New Zealand: longitudinal findings from the Health, Work and Retirement study. Int J Care Caring. 2017;1(3):309-29. doi: 10.1332/239788217X15040798472015.

18. Maidment J. Informal caregivers: An invisible unpaid workforce. In: Maidment J, Beddoe L, editors. Social policy for social work and human services in Aotearoa New Zealand: diverse perspectives. Christchurch: Canterbury University Press; 2016. p. 180-93.

19. Statistics New Zealand. Loneliness in New Zealand: findings from the 2010 NZ General Social Survey. Wellington, New Zealand: Statistics New Zealand, 2013.

20. Gott M, Allen R, Moeke-Maxwell T, Gardiner C, Robinson J. 'No matter what the cost': a qualitative study of the financial costs faced by family and whānau caregivers within a palliative care context. Palliat Med. 2015;29(6):518-28. Epub 2015/02/13. doi: 10.1177/0269216315569337. PubMed PMID: 25680378.

21. Alzheimer’s NZ. The Deloitte’s dementia economic impact report 2016. 2017.

22. Wimo A, Jönsson L, Bond J, Prince M, Winblad B. The worldwide economic impact of dementia 2010. Alzheimers Dement. 2013;9(1):1-11.e3. doi: 10.1016/j.jalz.2012.11.006. PubMed PMID: 23305821.

23. Orpin P, Stirling C, Hetherington S, Robinson A. Rural dementia carers: formal and informal sources of support. Ageing Soc. 2014;34(2):185-208. Epub 2012/08/15. doi:

10.1017/S0144686X12000827. PubMed PMID: 24453384.

24. Joling KJ, van Hout HP, Schellevis FG, van der Horst HE, Scheltens P, Knol DL, et al. Incidence of depression and anxiety in the spouses of patients with dementia: a naturalistic cohort study of recorded morbidity with a 6-year follow-up. Am J Geriatr Psychiatry. 2010;18(2):146-53. Epub 2010/01/28. doi: 10.1097/JGP.0b013e3181bf9f0f. PubMed PMID: 20104070.

25. Janssen EP, de Vugt M, Köhler S, Wolfs C, Kerpershoek L, Handels RL, et al. Caregiver profiles in dementia related to quality of life, depression and perseverance time in the European Actifcare study: the importance of social health. Aging Ment Health. 2017;21(1):49-57. Epub 2016/11/22. doi: 10.1080/13607863.2016.1255716. PubMed PMID: 27869480.

26. Isik AT, Soysal P, Solmi M, Veronese N. Bidirectional relationship between caregiver burden and neuropsychiatric symptoms in patients with Alzheimer's disease: a narrative review. Int J Geriatr Psychiatry. 2019;34(9):1326-34. Epub 2018/09/11. doi: 10.1002/gps.4965. PubMed PMID: 30198597.

27. Gibson R, Gander P. Factors associated with the sleep of carers: a survey of New Zealanders supporting a family member with cognitive impairment or dementia. Dementia (London). 2021;20(3):919-35. Epub 2020/04/02. doi: 10.1177/1471301220915071. PubMed PMID: 32228061.

28. von Kanel R, Mausbach BT, Dimsdale JE, Ziegler MG, Mills PJ, Allison MA, et al. Refining caregiver vulnerability for clinical practice: determinants of self-rated health in spousal dementia caregivers. BMC Geriatr. 2019;19(1):18. Epub 2019/01/24. doi: 10.1186/s12877-019-1033-2. PubMed PMID: 30669980; PubMed Central PMCID: PMC6343283.

29. Liu Y, Dokos M, Fauth EB, Lee YG, Zarit SH. Financial strain, employment, and role

captivity and overload over time among dementia family caregivers. Gerontologist. 2019;59(5):e512-e20. Epub 2019/07/20. doi: 10.1093/geront/gnz099. PubMed PMID: 31322654; PubMed Central PMCID: PMC6857684.

30. Feast A, Moniz-Cook E, Stoner C, Charlesworth G, Orrell M. A systematic review of the relationship between behavioral and psychological symptoms (BPSD) and caregiver well-being. Int Psychogeriatr. 2016;28(11):1761-74. Epub 2016/06/28. doi: 10.1017/S1041610216000922. PubMed PMID: 27345942.

31. Brown EE, Kumar S, Rajji TK, Pollock BG, Mulsant BH. Anticipating and mitigating the impact of the COVID-19 pandemic on Alzheimer's disease and related dementias. Am J Geriatr Psychiatry. 2020;28(7):712-21. Epub 2020/04/26. doi: 10.1016/j.jagp.2020.04.010. PubMed PMID: 32331845; PubMed Central PMCID: PMC7165101.

32. Navaie-Waliser M, Spriggs A, Feldman PH. Informal caregiving: differential experiences by gender. Med Care. 2002;40(12):1249-59. Epub 2002/11/30. doi: 10.1097/01.MLR.0000036408.76220.1F. PubMed PMID: 12458306.

33. Associate Minister of Health. Healthy ageing strategy. Wellington: Ministry of Health, 2016.

34. Ministry of Social Development, NZ Carers Alliance. Mahi aroha: Carers' strategy action plan 2019-2023. Wellington: Ministry of Social Development, 2019.

35. Ministry of Social Development. Carers' strategy and five-year action plan 2008. Wellington: Ministry of Social Development, 2008.

36. Australian Government. National carer strategy. Canberra: Commonwealth of Australia, 2011.

37. Carers UK. Helping families manage care – strategy 2011-2014. London: Carers UK, 2010.

38. Lamura G, Mnich E, Nolan M, Wojszel B, Krevers B, Mestheneos L, et al. Family carers' experiences using support services in Europe: empirical evidence from the EUROFAMCARE study. Gerontologist. 2008;48(6):752-71. Epub 2009/01/14. doi: 10.1093/geront/48.6.752. PubMed PMID: 19139249.

39. Criel B, Vanlerberghe V, De Koker B, Decraene B, Engels E, Waltens R. Informal home care for elderly in Belgium: a study on the features and challenges of informal care at local level. Community Ment Health J. 2014;50(7):848-53. Epub 2014/01/23. doi: 10.1007/s10597-014-9696-9. PubMed PMID: 24449429.

40. Williams LA, Moeke-Maxwell T, Wiles J, Black S, Trussardi G, Kerse N, et al. How family caregivers help older relatives navigate statutory services at the end of life: a descriptive qualitative study. Palliat Med. 2018;32(6):1124-32. Epub 2018/04/19. doi: 10.1177/0269216318765853. PubMed PMID: 29667475.

41. Ragnat A, Parsons, J., Radhakrishnan, R. The barriers determining the use of the carer support subsidy in the Northland region of New Zealand - a qualitative study. J Geriatr Care Res. 2018;5(1).

42. National Health Committee. Rural health: challenges of distance, opportunities for innovation. Wellington: Naional Health Committee, 2010.

43. Parsons M, Senior HE, Kerse N, Chen MH, Jacobs S, Vanderhoorn S, et al. The Assessment of Services Promoting Independence and Recovery in Elders trial (ASPIRE): a pre-planned meta-analysis of three independent randomised controlled trial evaluations of ageing in place initiatives in New Zealand. Age Ageing. 2012;41(6):722-8. Epub 2012/08/25. doi: 10.1093/ageing/afs113. PubMed PMID: 22918089.

44. Larkin M, Milne A. Carers and empowerment in the UK: a critical reflection. Soc Policy Soc. 2014;13(1):25-38. doi: 10.1017/S1474746413000262. PubMed PMID:

1467633599.
45. Greenwood N, Habibi R, Smith R, Manthorpe J. Barriers to access and minority ethnic carers' satisfaction with social care services in the community: a systematic review of qualitative and quantitative literature. Health Soc Care Community. 2015;23(1):64-78. Epub 2014/08/20. doi: 10.1111/hsc.12116. PubMed PMID: 25135207; PubMed Central PMCID: PMC4283974.
46. Tamasese TK, Parsons TL, Waldegrave C. Pacific perspectives on ageing in New Zealand. Pacific focused qualitative research prepared for the New Zealand Longitudinal Study of Ageing (NZLSA). Lower Hutt, NZ: The Family Centre March, 2014.
47. Latu M. The invisible caregiver: challenges of Tongan Family caregivers for the elderly in Auckland New Zealand: University of Auckland; 2015.
48. Mead H. Tiaknga Māori: Living by Māori values. Wellington: Huia; 2003.
49. Taskforce on Whānau-Centred Initiatives. Whānau ora: Report of the Taskforce on Whānau-Centred Initiatives. Wellington: Taskforce on Whānau-Centred Initiatives, 2010.
50. Collins A, Wilson G. Māori and informal caregiving a background paper prepared for the National Health Committee. Wellington: Māori Development Research Centre, Ministry of Health, 2008.
51. Dudley M, Menzies O, HElder H, Nathan L, Garrett N, Wilson D. Mate wareware: understanding 'dementia' from a Māori perspective. N Z Med J. 2019;132(1503):66-74.
52. Nathan LK. Mai ngā reo pou tangata. Hamilton: The University of Waikato; 2019.
53. Blakely T, Ajwani S, Robson B, Tobias M, Bonne M. Decades of disparity: widening ethnic mortality gaps from 1980 to 1999. N Z Med J. 2004;117(1199):U995. Epub 2004/10/12. PubMed PMID: 15475978.

54. Davis P, Lay-Yee R, Dyall L, Briant R, Sporle A, Brunt D, et al. Quality of hospital care for Māori patients in New Zealand: retrospective cross-sectional assessment. Lancet. 2006;367(9526):1920-5. Epub 2006/06/13. doi: 10.1016/s0140-6736(06)68847-8. PubMed PMID: 16765761.
55. Ministry of Health, University of Otago. Decades of disparity III: ethnic and socio-economic inequalities in mortality, New Zealand 1981-1999. Wellington: Ministry of Health, 2006.
56. Blakely T, Tobias M, Atkinson J, Yeh L-C, Huang K. Tracking Disparity: Trends in ethnic and socioeconomic inequalities in mortality, 1981–2004. Wellington: Ministry of Health, 2007.
57. Waikato District Health Board. Reducing inequalities action plan 2007-2010. Hamilton: Waikato District Health Board, 2007.
58. Lay-Yee R, Pearson J, Davis P, von Randow M, Kerse N, Brown L. Changing the balance of social care for older people: simulating scenarios under demographic ageing in New Zealand. Health Soc Care Community. 2017;25(3):962-74. Epub 2016/10/07. doi: 10.1111/hsc.12394. PubMed PMID: 27709717.
59. Kuluski K, Peckham A, Gill A, Arneja J, Morton-Chang F, Parsons J, et al. "You've got to look after yourself, to be able to look after them" a qualitative study of the unmet needs of caregivers of community based primary health care patients. BMC Geriatr. 2018;18(1):275. Epub 2018/11/14. doi: 10.1186/s12877-018-0962-5. PubMed PMID: 30419819; PubMed Central PMCID: PMC6233534.
60. Wong-Cornall C, Parsons J, Sheridan N, Kenealy T, Peckham A. Extending "continuity of care" to include the contribution of family carers. Int J Integr Care. 2017;17(2):11. Epub 2017/10/04. doi: 10.5334/ijic.2545. PubMed PMID: 28970752; PubMed Central PMCID: PMC5624104.

61. Goodhead A, McDonald J. Informal caregivers literature review: a report prepared for the National Health Committee. Wellington: Health Services Research Centre, Victoria University of Wellington, 2007.

62. National Health Board. Faiva ora national Pasifika disability plan 2010–2013. Wellington: Ministry of Health, 2010.

63. Minister of Health. Korero marama: health literacy and Māori results from the 2006 adult literacy and life skills survey. Wellington: Ministry of Health, 2010.

64. Health Partners Consulting Group. Metro-Auckland Pacific population health profile. Auckland: Health Partners Consulting Group, 2012.

65. Ministry of Health. Tupu Ola Moui: Pacific health chart book 2012. Wellington: Ministry of Health, 2012.

66. Harris R, Cormack D, Tobias M, Yeh L-C, Talamaivao N, Minster J, et al. Self-reported experience of racial discrimination and health care use in New Zealand: results from the 2006/07 New Zealand Health Survey. Am J Pub Health. 2012;102(5):1012-9. Epub 2012/03/15. doi: 10.2105/AJPH.2011.300626. PubMed PMID: 22420811.

67. Neufeld A, Harrison MJ, Stewart MJ, Hughes KD, Spitzer D. Immigrant women: making connections to community resources for support in family caregiving. Qual Health Res. 2002;12(6):751-68. Epub 2002/07/12. doi: 10.1177/104973230201200603. PubMed PMID: 12109721.

68. Simpson ML, Berryman K, Oetzel J, Iti T, Reddy R. A cultural analysis of New Zealand palliative care brochures. Health Promot Int. 2016;31(4):839-48. Epub 2015/07/15. doi: 10.1093/heapro/dav067. PubMed PMID: 26163520.

69. Oetzel JG, Simpson M, Berryman K, Reddy R. Differences in ideal communication behaviours during end-of-life care for Maori carers/patients and palliative care workers. Palliat Med. 2015;29(8):764-6. Epub 2015/04/22. doi: 10.1177/0269216315583619.

PubMed PMID: 25895535.
70. Stephens C, Budge C, Alpass F, Stevenson B. The social networks, digital media use and barriers to participation of older people in rural New Zealand: differences across regions. 67th Annual Scientific Meeting "Making Connections: From Cells to Societies" November 5–9, 2014 The Gerontologist. 2014;54:445. doi: 10.1093/geront/gnu106.
71. Lilley R, de Graaf B, Kool B, Davie G, Reid P, Dicker B, et al. Geographical and population disparities in timely access to prehospital and advanced level emergency care in New Zealand: a cross-sectional study. BMJ Open. 2019;9(7):e026026. Epub 2019/07/28. doi: 10.1136/bmjopen-2018-026026. PubMed PMID: 31350239; PubMed Central PMCID: PMC6661642.
72. Citizens Advice Bureaux New Zealand. Face to face with digital exclusion: A CAB spotlight report into the impacts of digital public services on inclusion and wellbeing. Wellington: Citizens Advice Bureaux, 2020.
73. Hanratty B, Drever F, Jacoby A, Whitehead M. Retirement age caregivers and deprivation of area of residence in England and Wales. Eur J Ageing. 2007;4(1):35-43. doi: 10.1007/s10433-007-0044-6. PubMed PMID: 28794770.
74. Wiles J, Moeke-Maxwell T, Williams L, Black S, Trussardi G, Gott M. Caregivers for people at end of life in advanced age: knowing, doing and negotiating care. Age Ageing. 2018;47(6):887-95. Epub 2018/08/31. doi: 10.1093/ageing/afy129. PubMed PMID: 30165558.
75. Morita A, Takano T, Nakamura K, Kizuki M, Seino K. Contribution of interaction with family, friends and neighbours, and sense of neighbourhood attachment to survival in senior citizens: 5-year follow-up study. Soc Sci Med. 2010;70(4):543-9. Epub 2009/12/01. doi: 10.1016/j.socscimed.2009.10.057. PubMed PMID: 19944506.

76. Gardner PJ. Natural neighborhood networks - important social networks in the lives of older adults aging in place. J Aging Stud. 2011;25(3):263-71. doi: 10.1016/j.jaging.2011.03.007. PubMed PMID: WOS:000292664400009.
77. Wiles J. Daily geographies of caregivers: mobility, routine, scale. Soc Sci Med. 2003;57(7):1307-25. Epub 2003/08/06. doi: 10.1016/s0277-9536(02)00508-7. PubMed PMID: 12899912.
78. Papastavrou E, Andreou P, Middleton N, Tsangari H, Papacostas S. Dementia caregiver burden association with community participation aspect of social capital. J Adv Nurs. 2015;71(12):2898-910. Epub 2015/09/09. doi: 10.1111/jan.12762. PubMed PMID: 26345604.
79. Winterton R, Warburton J. Does place matter? Reviewing the experience of disadvantage for older people in rural Australia. Rural Society. 2014;20(2):187-97. doi: 10.5172/rsj.20.2.187.
80. Hanlon N, Kearns R. Health and Rural Places. In: Shucksmith M, Brown D, editors. Routledge international handbook of rural studies. London: Routledge; 2016. p. 92-100.
81. Skinner MW, Rosenberg MW, Lovell SA, Dunn JR, Everitt JC, Hanlon N, et al. Services for seniors in small-town Canada: the paradox of community. Can J Nurs Res. 2008;40(1):81-101. Epub 2008/05/08. PubMed PMID: 18459273.
82. Joseph A, Cloutier-Fisher D. Ageing in rural communities: vulnerable people in vulnerable places. In: Andrews G, Phillips D, editors. Ageing and place. London: Routledge; 2005. p. 149-62.
83. Knauss J, Moyer D. The role of advocacy in our adventure with Alzheimer's. Dementia (London). 2006;5(1):67-72. doi: 10.1177/1471301206059755.
84. Levine C, Halper D, Peist A, Gould DA. Bridging troubled waters: family caregivers, transitions, and long-term care. Health Aff (Millwood). 2010;29(1):116-24. Epub

2010/01/06. doi: 10.1377/hlthaff.2009.0520. PubMed PMID: 20048369.

85. Clemmer SJ, Ward-Griffin C, Forbes D. Family members providing home-based palliative care to older adults: the enactment of multiple roles. Can J Aging. 2008;27(3):267-83. Epub 2009/01/23. doi: 10.3138/cja.27.3.267. PubMed PMID: 19158043.

86. Kuluski K, Peckham A, Gill A, Gagnon D, Wong-Cornall C, McKillop A, et al. What is important to older people with multimorbidity and their caregivers? Identifying attributes of person centered care from the user perspective. Int J Integr Care. 2019;19(3):4. Epub 2019/08/02. doi: 10.5334/ijic.4655. PubMed PMID: 31367203; PubMed Central PMCID: PMC6659759.

87. Breton M, Gray CS, Sheridan N, Shaw J, Parsons J, Wankah P, et al. Implementing community based primary healthcare for older adults with complex needs in Quebec, Ontario and New-Zealand: describing nine cases. Int J Integr Care. 2017;17(2):12. Epub 2017/10/04. doi: 10.5334/ijic.2506. PubMed PMID: 28970753; PubMed Central PMCID: PMC5624082.

88. Kuluski K, Gill A, McKillop A, Parsons J, Peckham A, Sheridan N, et al. The Unmet Needs of Patients and Carers within Community Based Primary Health Care. Int J Integr Care. 2017;17(3). doi: 10.5334/ijic.3251. PubMed PMID: WOS:000565199000140.

89. Burholt V, Sardani AV. The impact of residential immobility and population turnover on the support networks of older people living in rural areas: evidence from CFAS Wales. Population, Space and Place. 2017;24(4S):e2132. doi: 10.1002/psp.2132.

90. Neufeld A, Harrison MJ, Stewart M, Hughes K. Advocacy of women family caregivers: response to nonsupportive interactions with professionals. Qual Health Res. 2008;18(3):301-10. Epub 2008/02/01. doi: 10.1177/1049732307313768. PubMed

PMID: 18235154.

91. Rea H, Kenealy T, Sheridan N, Gorman D. Invisible care: do we need a Code of Rights to protect family and informal carers. N Z Med J. 2010;123(1317):5-6.

92. Arksey H, Hirst M. Unpaid carers' access to and use of primary care services. Prim Health Care Res Dev. 2005;6(2):101-16. doi: 10.1191/1463423605pc230oa.

93. Donelan K, Hill CA, Hoffman C, Scoles K, Feldman PH, Levine C, et al. Challenged to care: informal caregivers in a changing health system. Health Aff (Millwood). 2002;21(4):222-31. Epub 2002/07/16. doi: 10.1377/hlthaff.21.4.222. PubMed PMID: 12117133.

94. Sheridan N, Kenealy T, Kuluski K, McKillop A, Parsons J, Wong-Cornall C. Are patient and carer experiences mirrored in the Practice Reviews of Self-management Support (PRISMS) provider taxonomy? Int J Integr Care. 2017;17(2):8. Epub 2017/10/04. doi: 10.5334/ijic.2483. PubMed PMID: 28970749; PubMed Central PMCID: PMC5624079.

95. Sheridan N, Kenealy T, Stewart L, Lampshire D, Robust TT, Parsons J, et al. When equity is central to research: implications for researchers and consumers in the research team. Int J Integr Care. 2016;17(2):14. Epub 2017/10/04. doi: 10.5334/ijic.2512. PubMed PMID: 28970755; PubMed Central PMCID: PMC5624117.

96. New Zealand Dementia Cooperative, Alzheimer's New Zealand, Dementia New Zealand. Improving dementia services in New Zealand - dementia action plan 2020-2025. Wellington, New Zealand: New Zealand Dementia Cooperative, Alzheimer's New Zealand, Dementia New Zealand, 2020.

97. Jorgensen D, Parsons M, Jacobs S, Arksey H. The New Zealand informal caregivers and their unmet need. N Z Med J. 2010;123 (1317):9-16.

98. King AI, Parsons M, Robinson E, Jorgensen D. Assessing the impact of a restorative

home care service in New Zealand: a cluster randomised controlled trial. Health Soc Care Community. 2012;20(4):365-74. Epub 2011/11/24. doi: 10.1111/j.1365-2524.2011.01039.x. PubMed PMID: 22106952.

99. Tremont G, Davis JD, Ott BR, Galioto R, Crook C, Papandonatos GD, et al. Randomized trial of the family intervention: telephone tracking-caregiver for dementia caregivers: use of community and healthcare resources. J Am Geriatr Soc. 2017;65(5):924-30. Epub 2016/12/23. doi: 10.1111/jgs.14684. PubMed PMID: 28008609; PubMed Central PMCID: PMC5510656.

100. King A, Parsons M. An evaluation of two respite models for older people and their informal caregivers. N Z Med J. 2005;118(1214):U1440. Epub 2005/05/12. PubMed PMID: 15886735.

101. Wiles J. Informal caregivers' experiences of formal support in a changing context. Health Soc Care Community. 2003;11(3):189-207. Epub 2003/06/26. doi: 10.1046/j.1365-2524.2003.00419.x. PubMed PMID: 12823424.

102. Wang YN, Shyu YI, Chen MC, Yang PS. Reconciling work and family caregiving among adult-child family caregivers of older people with dementia: effects on role strain and depressive symptoms. J Adv Nurs. 2011;67(4):829-40. Epub 2010/11/17. doi: 10.1111/j.1365-2648.2010.05505.x. PubMed PMID: 21077933.

103. Edwards AB, Zarit SH, Stephens MA, Townsend A. Employed family caregivers of cognitively impaired elderly: an examination of role strain and depressive symptoms. Aging Ment Health. 2002;6(1):55-61. Epub 2002/02/06. doi: 10.1080/13607860120101149. PubMed PMID: 11827623.

104. Stephens C, Alpass F, Stevenson B. Inclusion, contribution and connection: a study of the ageing in Aotearoa. Summary Report Palmerston North: Massey University 2014.

105. Covinsky KE, Eng C, Lui L-Y, Sands LP, Sehgal AR, Walter LC, et al. Reduced

employment in caregivers of frail elders: impact of ethnicity, patient clinical characteristics, and caregiver characteristics. J Gerontol A Biol Sci Med Sci. 2001;56(11):M707-M13. Epub 2001/10/30. doi: 10.1093/gerona/56.11.M707. PubMed PMID: 11682579.

106. Alpass F, Keeling S, Allen J, Stevenson B, Stephens C. Reconciling work and caregiving responsibilities among older workers in New Zealand. J Cross Cult Gerontol. 2017;32(3):323-37. Epub 2017/07/01. doi: 10.1007/s10823-017-9327-3. PubMed PMID: 28664423.

107. Bijnsdorp FM, Pasman HRW, Francke AL, Evans N, Peeters CFW, Broese van Groenou MI. Who provides care in the last year of life? A description of care networks of community-dwelling older adults in the Netherlands. BMC Palliat Care. 2019;18(1):41. Epub 2019/05/17. doi: 10.1186/s12904-019-0425-6. PubMed PMID: 31092227; PubMed Central PMCID: PMC6521417.

108. Jacobs MT, Broese van Groenou MI, Aartsen MJ, Deeg DJH. Diversity in Older adults' care networks: the added value of individual beliefs and social network proximity. J Gerontol B Psychol Sci Soc Sci. 2018;73(2):326-36. Epub 2016/02/26. doi: 10.1093/geronb/gbw012. PubMed PMID: 26912490.

109. Keating N, Dosman D. Social capital and the care networks of frail seniors. Can Rev Sociol. 2009;46(4):301-18. Epub 2010/05/21. doi: 10.1111/j.1755-618x.2009.01216.x. PubMed PMID: 20481408.

110. Burholt V, Dobbs C, Victor C. Social support networks of older migrants in England and Wales: the role of collectivist culture. Ageing Soc. 2017;38(7):1453-77. doi: 10.1017/s0144686x17000034.

111. Wenger GC, Burholt V, Scott A. Dementia and help with household tasks: a comparison of cases and non-cases. Health Place. 1998;4(1):33-44. Epub 2000/02/12.

doi: 10.1016/s1353-8292(97)00024-5. PubMed PMID: 10671009.

112. Covinsky KE, Newcomer R, Fox P, Wood J, Sands L, Dane K, et al. Patient and caregiver characteristics associated with depression in caregivers of patients with dementia. Journal of general internal medicine. 2003;18(12):1006-14. doi: 10.1111/j.1525-1497.2003.30103.x. PubMed PMID: 14687259.

113. Sherman CW, Webster NJ, Antonucci TC. Dementia Caregiving in the Context of Late-Life Remarriage: Support Networks, Relationship Quality, and Well-being. Journal of Marriage and Family. 2013;75(5):1149-63. doi: 10.1111/jomf.12059. PubMed PMID: WOS:000323829900007.

114. Lien YF, Huang HM. Challenges in intergenerational caregiving for frail older people: A multiple case study. Nurs Health Sci. 2017;19(1):81-7. Epub 2016/12/28. doi: 10.1111/nhs.12314. PubMed PMID: 28026105.

115. Xu J, Liu PJ, Beach S. Multiple Caregivers, Many Minds: Family Discord and Caregiver Outcomes. Gerontologist. 2021;61(5):661-9. Epub 2020/06/17. doi: 10.1093/geront/gnaa067. PubMed PMID: 32539097.

116. Mosquera I, Vergara I, Larranaga I, Machon M, del Rio M, Calderon C. Measuring the impact of informal elderly caregiving: a systematic review of tools. Qual Life Res. 2016;25(5):1059-92. Epub 2015/10/18. doi: 10.1007/s11136-015-1159-4. PubMed PMID: 26475138.

117. Brown RM, Brown SL. Informal caregiving: a reappraisal of effects on caregivers. Soc Issues Policy Rev. 2014;8(1):74-102. doi: 10.1111/sipr.12002. PubMed PMID: WOS:000329569200003.

118. National Academies of Sciences, Engineering, & Medicine. Families caring for an aging America. Washington DC: National Academies Press, 2016.

119. Bom J, Bakx P, Schut F, van Doorslaer E. The impact of informal caregiving for older

adults on the health of various types of caregivers: a systematic review. Gerontologist. 2019;59(5):e629-e42. doi: 10.1093/geront/gny137. PubMed PMID: 30395200; PubMed Central PMCID: PMC6850889.

120. Swain N. Distress in informal carers of the elderly in New Zealand. N Z Med J. 2018;131(1485).

121. Gardiner C, Brereton L, Frey R, Wilkinson-Meyers L, Gott M. Exploring the financial impact of caring for family members receiving palliative and end-of-life care: a systematic review of the literature. Palliat Med. 2014;28(5):375-90. Epub 2013/11/10. doi: 10.1177/0269216313510588. PubMed PMID: 24201134.

122. Robison J, Fortinsky R, Kleppinger A, Shugrue N, Porter M. A broader view of family caregiving: effects of caregiving and caregiver conditions on depressive symptoms, health, work, and social isolation. J Gerontol B Psychol Sci Soc Sci. 2009;64(6):788-98. doi: 10.1093/geronb/gbp015. PubMed PMID: 19318470.

123. Horrell B, Stephens C, Breheny M. Capability to care: Supporting the health of informal caregivers for older people. Health Psychol. 2015;34(4):339-48. Epub 2014/08/12. doi: 10.1037/hea0000144/. PubMed PMID: 25110849.

124. Carbonneau H, Caron C, Desrosiers J. Development of a conceptual framework of positive aspects of caregiving in dementia. Dementia (London). 2010;9(3):327-53. doi: 10.1177/1471301210375316.

125. Carers New Zealand, New Zealand Carers Alliance. Caring in lockdown: forgotten families during COVID-19. Auckland: Carers New Zealand, 2020.

126. Phillips D, Paul G, Fahy M, Dowling-Hetherington L, Kroll T, Moloney B, et al. The invisible workforce during the COVID-19 pandemic:fFamily carers at the frontline. HRB Open Res. 2020;3:24-. doi: 10.12688/hrbopenres.13059.1. PubMed PMID: 32551415.

127. Budnick A, Hering C, Eggert S, Teubner C, Suhr R, Kuhlmey A, et al. Informal caregivers during the COVID-19 pandemic perceive additional burden: findings from an ad-hoc survey in Germany. BMC Health Serv Res. 2021;21(1):353. Epub 2021/04/16. doi: 10.1186/s12913-021-06359-7. PubMed PMID: 33863337; PubMed Central PMCID: PMC8050992.

128. Chan EYY, Gobat N, Kim JH, Newnham EA, Huang Z, Hung H, et al. Informal home care providers: the forgotten health-care workers during the COVID-19 pandemic. Lancet. 2020;395(10242):1957-9. doi: 10.1016/S0140-6736(20)31254-X. PubMed PMID: 32497509; PubMed Central PMCID: PMC7263813.

129. Sevenhuijsen S. The place of care: The relevance of the feminist ethic of care for social policy. Feminist Theory. 2003;4(2):179-97. doi: 10.1177_14647001030042006.

130. Skinner M, Hanlon N. Ageing resource communities: new frontiers of rural population change, community development and voluntarism. Oxford: Routledge; 2016.

131. Carers NSW Australia. The COVID-19 pandemic: challenges and oppotunities for carers in NSW. Sydney, Australia: Carers NSW Australia, 2020.

132. Sylvester A, Toland J, Parore P. Is the digital divide still relevant in 2017? Two cases from marginalised communities in Aotearoa-New Zealand. PACIS 2017 Proceedings. 2017;123.

133. Carers UK. Caring behind closed doors - forgotten families in the coronavirus outbreak. London: Carers UK, 2020.

134. Stokes JE, Patterson SE. Intergenerational relationships, family caregiving policy, and COVID-19 in the United States. J Aging Soc Policy. 2020;32(4-5):416-24. doi: 10.1080/08959420.2020.1770031. PubMed PMID: 32489144; PubMed Central PMCID: PMC7754249.

135. Blume C, Schmidt MH, Cajochen C. Effects of the COVID-19 lockdown on human

sleep and rest-activity rhythms. Curr Biol. 2020;30(14):R795-R7. Epub 2020/06/10. doi: 10.1016/j.cub.2020.06.021. PubMed PMID: 32693067.

136. Wu B. Social isolation and loneliness among older adults in the context of COVID-19: a global challenge. Glob Health Res Policy. 2020;5(1):27. Epub 2020/06/10. doi: 10.1186/s41256-020-00154-3. PubMed PMID: 32514427; PubMed Central PMCID: PMC7272234.

137. Keating N, Phillips J. A critical human ecology perspective on rural ageing. In: Keating N, editor. Rural ageing: a good place to grow old? Bristol: Policy Press; 2008. p. 1-10.

138. Versey HS. Caregiving and women's health: toward an intersectional approach. Womens Health Issues. 2017;27(2):117-20. Epub 2017/03/05. doi: 10.1016/j.whi.2017.01.009. PubMed PMID: 28258803.

139. Burholt V, Scharf T. Critical social gerontology and rural ageing. In: Skinner M, Winterton R, Walsh K, editors. Rural gerontology: Towards critical perspectives on rural ageing. London and New York: Routledge; 2021.

140. Diez Roux AV. Complex systems thinking and current impasses in health disparities research. Am J Public Health. 2011;101(9):1627-34. Epub 2011/07/23. doi: 10.2105/AJPH.2011.300149. PubMed PMID: 21778505; PubMed Central PMCID: PMC3154209.

141. Powell T. Prudent healthcare: a turning point for health and care in Wales - Click on Wales: Institute of Welsh Affairs; 2017 [updated 2017-07-04; cited 2017 6 September]. Available from: http://www.iwa.wales/click/2017/07/prudent-healthcare-turning-point-health-care-wales/.

142. Stephens C, Burholt V, Keating N. Collecting qualitative data with older people. In: Flick U, editor. The SAGE handbook of qualitative data collection: Sage; 2017. p. 632-51.

143. Waterman H, Tillen D, Dickson R, de Koning K. Action research: a systematic review and guidance for assessment. Health technology assessment (Winchester, England). 2001;5(23):iii-157. Epub 2002/01/12. PubMed PMID: 11785749.

144. Kothari A, Wathen CN. A critical second look at integrated knowledge translation. Health Policy. 2013;109(2):187-91. Epub 2012/12/12. doi: 10.1016/j.healthpol.2012.11.004. PubMed PMID: 23228520.

145. Gagliardi AR, Berta W, Kothari A, Boyko J, Urquhart R. Integrated knowledge translation (IKT) in health care: a scoping review. Implement Sci. 2016;11(1):38. doi: 10.1186/s13012-016-0399-1. PubMed PMID: 26988000; PubMed Central PMCID: PMC4797171.

146. Tetroe J. Knowledge translation at the Canadian Institutes of Health Research: a primer. Focus Technical Brief. 2007;18:1-8.

147. Nomura M, Makimoto K, Kato M, Shiba T, Matsuura C, Shigenobu K, et al. Empowering older people with early dementia and family caregivers: a participatory action research study. Int J Nurs Stud. 2009;46(4):431-41. doi: 10.1016/j.ijnurstu.2007.09.009. PubMed PMID: 17983619.

148. Higginbottom G, Liamputtong P. Participatory qualitative research methodologies in health. London: Sage; 2015.

149. Patmore C, Qureshi H, Nicholas E. Consulting older community care clients about their services: some lessons for researchers and service managers. Research Policy and Planning: The Journal of the Social Services Research. 2008;18(1):4-11.

150. Davis SF, Silvester A, Barnett D, Farndon L, Ismail M. Hearing the voices of older adult patients: processes and findings to inform health services research. Res Involv Engagem. 2019;5(1):11. doi: 10.1186/s40900-019-0143-5. PubMed PMID: 30834143; PubMed Central PMCID: PMC6385442.

151. Archibald MM, Ambagtsheer RC, Casey MG, Lawless M. Using Zoom videoconferencing for qualitative data collection: perceptions and experiences of researchers and participants. International Journal of Qualitative Methods. 2019;18:1609406919874596. doi: 10.1177/1609406919874596. PubMed PMID: WOS:000485252700001.

152. Gott M, Moeke-Maxwell T, Morgan T, Black S, Williams L, Boyd M, et al. Working bi-culturally within a palliative care research context: the development of the Te Ārai Palliative Care and End of Life Research Group. Mortality. 2017;22(4):291-307. doi: 10.1080/13576275.2016.1216955.

153. National Institute for Health Research, Chief Scientist Office, Health and Care Research Wales, Public Health Agency. National standards for public involvement in research. London: NIHR Commissioning Facility, 2018.

154. Health and Social Care Research Wales. Guidance on funding and grant bid costings for public involvement activities. Cardiff, UK: Health and Social Care Research Wales, Welsh Government, 2019.

155. Mack N, Woodsong C, Macqueen KM, Gues, G, Namey E. Qualitative research methods: a data collector's field guide. North Carolina: Family Health International and US Agency for International Development; 2005.

156. Schutz A. The phenomenology of the social world. Evanston, IL: Northwestern University Press; 1967.

157. Ritchie J, Spencer L. Qualitative data analysis for applied policy research 2002 2020/05/27. In: The qualitative researcher's companion [Internet]. Thousand Oaks, California: Sage Publications Inc; [305-29]. Available from: https://methods.sagepub.com/book/the-qualitative-researchers-companion.

158. Ayres L, Kavanaugh K, Knafl KA. Within-case and across-case approaches to

qualitative data analysis. Qualitative Health Research. 2003;13(6):871-83. doi: 10.1177/1049732303013006008. PubMed PMID: 12891720.

159. Burholt V, Maruthakutti R, Maddock CA. A cultural framework of care and social protection for older people in India. GeroPsych. 2020:1-3. doi: 10.1024/1662-9647/a000251.

160. Polkinghorne DE. Narrative configuration in qualitative analysis. Int J Qual Stud Educ. 1995;8(1):5-23. doi: 10.1080/0951839950080103.

161. Hunter A, Lusardi P, Zucker D, Jacelon C, Chandler G. Making meaning: the creative component in qualitative research. Qual Health Res. 2002;12(3):388-98. doi: 10.1177/104973202129119964. PubMed PMID: 11918103.

162. Halcomb EJ, Davidson PM. Is verbatim transcription of interview data always necessary? Appl Nurs Res. 2006;19(1):38-42. doi: 10.1016/j.apnr.2005.06.001. PubMed PMID: 16455440.

163. Thomas DR. Feedback from research participants: are member checks useful in qualitative research? Qual Res Psychol. 2017;14(1):23-41. doi: 10.1080/14780887.2016.1219435.

164. Brandon PR, Fukunaga LL. The state of the empirical research literature on stakeholder involvement in program evaluation. Am J Eval. 2013;35(1):26-44. doi: 10.1177/1098214013503699.

165. Tan L, Szebeko D. Co-designing for dementia: The Alzheimer 100 project. Australas Med J. 2009;1(12):185-98. doi: 10.4066/AMJ.2009.97.

166. Gubrium A. Digital storytelling: an emergent method for health promotion research and practice. Health Promot Pract. 2009;10(2):186-91. Epub 2009/04/18. doi: 10.1177/1524839909332600. PubMed PMID: 19372280.

167. Hardy P, Sumner T, editors. Cultivating compassion: how digital storytelling is

transforming healthcare. 2nd ed: Palgrave MacMillan; 2018.

168. Ministry of Health. National Health Emergency Plan: A framework for the health and disability sector. Wellington: Ministry of Health, 2015.

Fig1. Human ecological model comprising inter-related systems that influence caregiving

Click here to access/download;Figure;Figure 1 (final).bmp

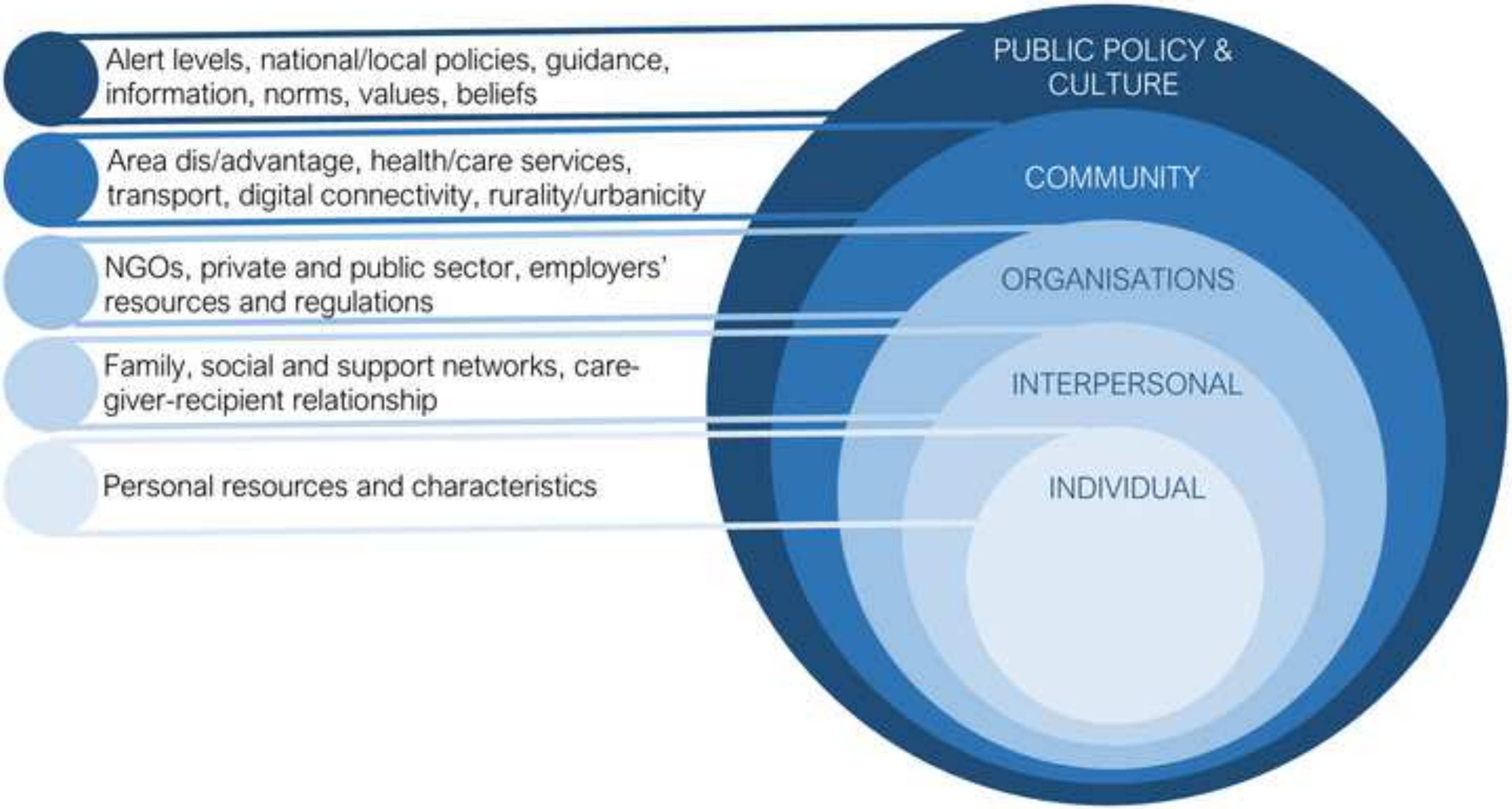

S1 Appendix, Demographic Questions: Caregivers

Click here to access/download
Supporting Information
S1 Appendix.pdf

S2 Appendix, Semi-Structured Interview Guide for Individual Caregivers

Click here to access/download
Supporting Information
S2 Appendix.pdf

S3 Appendix, Semi-Structured Interview Guide for Groups (>1 Caregiver)

Click here to access/download
Supporting Information
S3 Appendix.pdf

S4 Appendix, Semi-Structured Interview Guide for Service Providers

Click here to access/download
Supporting Information
S4 Appendix.pdf
